# Durability analysis of the highly effective BNT162b2 vaccine against COVID-19

**DOI:** 10.1093/pnasnexus/pgac082

**Published:** 2022-06-08

**Authors:** Arjun Puranik, Patrick J Lenehan, John C O'Horo, Colin Pawlowski, Michiel J M Niesen, Abinash Virk, Melanie D Swift, Walter Kremers, A J Venkatakrishnan, Joel E Gordon, Holly L Geyer, Leigh Lewis Speicher, Venky Soundararajan, Andrew D Badley

**Affiliations:** nference, Cambridge, MA 02139, USA; nference, Cambridge, MA 02139, USA; Division of Infectious Diseases, Mayo Clinic, Rochester, MN 55902, USA; Division of Pulmonary and Critical Care Medicine, Mayo Clinic, Rochester, MN 55902, USA; nference, Cambridge, MA 02139, USA; nference, Cambridge, MA 02139, USA; Division of Infectious Diseases, Mayo Clinic, Rochester, MN 55902, USA; Division of Aerospace, Occupational and Preventive Medicine, Mayo Clinic, Rochester, MN 55902, USA; Division of Biomedical Statistics, Mayo Clinic, Rochester, MN 55902, USA; nference, Cambridge, MA 02139, USA; Department of Family Medicine, Mayo Clinic Health System, Mankato, MN 56001, USA; Division of Hospital Internal Medicine, Mayo Clinic, Scottsdale, AZ 85259, USA; Division of General Internal Medicine, Mayo Clinic, Jacksonville, FL 32224, USA; nference, Cambridge, MA 02139, USA; nference Labs, Bengaluru, Karnataka 560017, India; Division of Infectious Diseases, Mayo Clinic, Rochester, MN 55902, USA; Department of Molecular Medicine, Mayo Clinic, Rochester, MN 55902, USA

**Keywords:** COVID-19, SARS-CoV-2, BNT162b2, Comirnaty, vaccine durability

## Abstract

COVID-19 vaccines are effective, but breakthrough infections have been increasingly reported. We conducted a test-negative case-control study to assess the durability of protection after full vaccination with BNT162b2 against polymerase chain reaction (PCR)-confirmed symptomatic SARS-CoV-2 infection, in a national medical practice from January 2021 through January 2022. We fit conditional logistic regression (CLR) models stratified on residential county and calendar time of testing to assess the association between time elapsed since vaccination and the odds of symptomatic infection or non-COVID-19 hospitalization (negative control), adjusted for several covariates. There were 5,985 symptomatic individuals with a positive test after full vaccination with BNT162b2 (cases) and 32,728 negative tests contributed by 27,753 symptomatic individuals after full vaccination (controls). The adjusted odds of symptomatic infection were higher 250 days after full vaccination versus at the date of full vaccination (Odds Ratio [OR]: 3.62, 95% CI: 2.52 to 5.20). The odds of infection were still lower 285 days after the first BNT162b2 dose as compared to 4 days after the first dose (OR: 0.50, 95% CI: 0.37 to 0.67), when immune protection approximates the unvaccinated status. Low rates of COVID-19 associated hospitalization or death in this cohort precluded analyses of these severe outcomes. The odds of non-COVID-19 associated hospitalization (negative control) decreased with time since vaccination, suggesting a possible underestimation of waning protection by this approach due to confounding factors. In summary, BNT162b2 strongly protected against symptomatic SARS-CoV-2 infection for at least 8 months after full vaccination, but the degree of protection waned significantly over this period.

Significance Statement The occurrence of SARS-CoV-2 infections in fully vaccinated individuals (“breakthrough infections”) highlights the importance of assessing how vaccine effectiveness changes over time in the context of highly transmissible and immune evasive SARS-CoV-2 lineages. Here, we assess the durability of protection conferred by BNT162b2 against symptomatic COVID-19 from January 2021 through January 2022. BNT162b2 provided strong protection for at least 8 months after full vaccination, but its effectiveness significantly waned during this period. This study highlights the importance of continuing to monitor the effectiveness and durability of protection conferred by COVID-19 vaccination series.

## Introduction

Infection with severe acute respiratory syndrome coronavirus 2 (SARS-CoV-2) and the resulting coronavirus disease-2019 (COVID-19) have impacted nearly 500 million people worldwide, resulting in more than 6.1 million deaths to date (1). Efforts were rapidly initiated during the early months of the pandemic to develop vaccines that would reduce community transmission and disease burden, leading to the clinical testing and subsequent Emergency Use Authorization (EUA) by the US Food and Drug Administration (FDA) of three vaccines by February 2021: BNT162b2 (mRNA vaccine by Pfizer-BioNTech authorized in December 2020), mRNA-1273 (mRNA vaccine by Moderna authorized in December 2020), and Ad26.COV2.S (adenoviral vector vaccine by Janssen authorized in February 2021) (2–6). As of 2022 April 8, over 565 million vaccine doses have been administered to over 255 million people in the United States, with over 75% of the adult population reaching full vaccination status per the Centers for Disease Control and Prevention (CDC) definition (7).

Randomized phase 3 clinical trials demonstrated over 90% efficacy in preventing symptomatic infection for both mRNA vaccines and approximately 65% efficacy in the same for Ad26.COV2.S (3, 5, 6). Subsequent analyses of individuals vaccinated outside the trial setting have yielded similar results, and BNT162b2 was granted full approval by the FDA for individuals 16 years of age and older in August 2021 (8–11). However, especially with the continued evolution of new SARS-CoV-2 strains, including the Delta and Omicron variants, it is important to assess vaccine effectiveness (VE) over time. Indeed, we and others reported lower levels of protection against infection during Delta variant surges in the summer and fall of 2021 (12–24), although it was generally not clear whether this change should be attributed to waning immunity over time after vaccination or other factors such as altered utilization of nonpharmaceutical interventions (e.g. masking, social distancing, and travel restrictions) or more efficient immune evasion by the Delta variant. Nevertheless, these signals prompted the recommendation of COVID-19 vaccine booster doses for adults in the United States and elsewhere (25, 26). Here, we use a modified test-negative case-control design to assess the durability of protection conferred by BNT162b2 among individuals who were vaccinated and subsequently tested for suspected SARS-CoV-2 infection at the Mayo Clinic.

## Results

### Primary analysis: change in the odds of symptomatic infection over time after full vaccination

**Figure 1.**
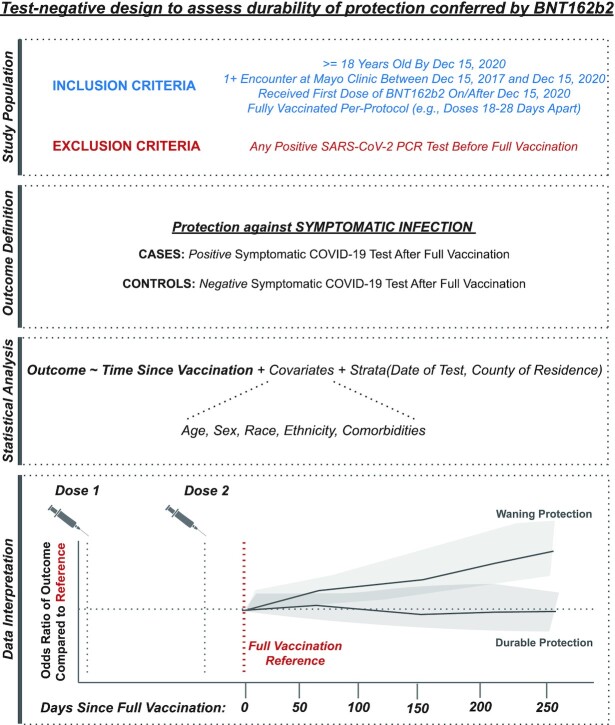
Schematic representation of study design. From top to bottom, (i) inclusion and exclusion criteria to define the population eligible for this test-negative analysis, (ii) definition of the clinical outcomes of interest, (iii) framework for statistical analysis, and (iv) schematic representation of data interpretation. (i) Individuals were included if they were at least 18 years old, had a record of at least one encounter at the Mayo Clinic in the 3 years prior to the study start date, and were fully vaccinated per-protocol with BNT162b2 (with the first dose administered on or after 2020 December 15), and underwent at least one symptomatic SARS-CoV-2 PCR test after the date of full vaccination. Individuals were excluded if they received a positive PCR test prior to their date of full vaccination. (ii) The outcome was defined as symptomatic SARS-CoV-2 infection, and cases and controls were defined accordingly. (iii) Conditional logistic regression was used to assess the potential relationship between the odds of experiencing symptomatic infection and time since vaccination, while accounting for other clinical and demographic covariates. (iv) The odds of symptomatic infection were assessed over time after full vaccination (modeled as a linear spline) relative to the odds at the date of full vaccination, which is expected to correspond to maximal vaccine-mediated protection. An increase in the odds ratio with time since vaccination would be interpreted as evidence for waning protection, while a consistent odds ratio over (relative) time would be interpreted as durable protection.

Of 219,399 individuals who received two doses of BNT162b2 18 to 28 days apart with no evidence of SARS-CoV-2 infection before reaching their date of full vaccination, 38,596 subsequently underwent symptomatic testing and were eligible for inclusion in the primary test-negative case-control analysis (Figure 1; Figure S1, Supplementary Material). There were 6,081 individuals who presented with positive tests (eligible cases) and 41,379 total negative tests (eligible controls) from 33,771 individuals (Figure S1 and Table S1, Supplementary Material). Cases and controls were generally similar in age, sex, race, ethnicity, and comorbidities to the underlying population of fully vaccinated individuals (Table S1, Supplementary Material). For the primary analysis, there were 5,985 cases and 32,728 controls that contributed to analyzable strata (i.e. strata with at least one case and at least one control; Figure S1 and Table S1, Supplementary Material).

**Figure 2.**
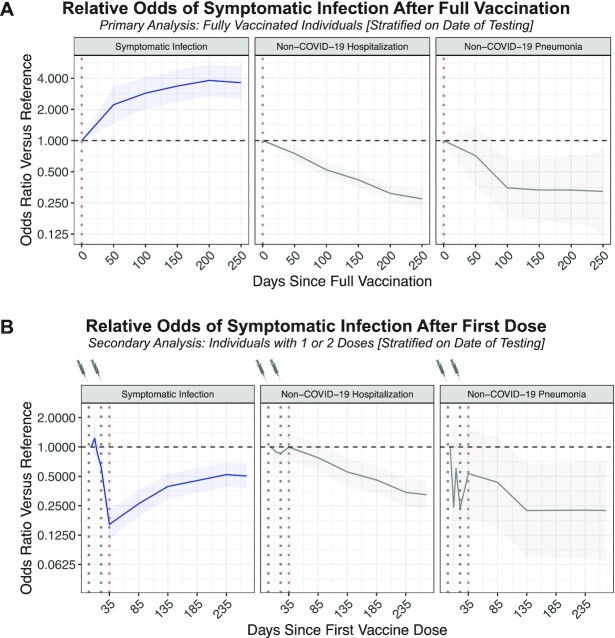
Adjusted odds for each outcome of interest over time relative to the date of full vaccination or the first vaccine dose. (A) In the primary analysis, the odds of each outcome were assessed with respect to time since full vaccination. The outcomes are symptomatic SARS-CoV-2 infection (blue; 5,985 cases), non-COVID-19 hospitalization (gray in middle; 5,599 cases), and non-COVID-19 pneumonia (gray on right; 447 cases). Each curve indicates the adjusted odds ratio comparing the odds of experiencing the outcome at the given time compared to at the time of full vaccination (Day 0 on this plot), which is expected to correspond to maximal vaccine-mediated protection. (B) In a secondary analysis, the odds of each outcome were assessed with respect to time since the first vaccine dose. Again, the outcomes are symptomatic SARS-CoV-2 infection (blue; 6,605 cases), non-COVID-19 hospitalization (gray in middle; 6,441 cases), and non-COVID-19 pneumonia (gray on right; 488 cases). Each curve indicates the adjusted odds ratio comparing the odds of experiencing the outcome at the given time versus at 4 days after the first dose (“reference”), which is expected to approximate an unvaccinated state. In both panels, the shaded regions indicate the 95% CI of the odds ratios.

Adjusted for age, sex, race, ethnicity, comorbidities, county, and the calendar date of testing, the odds of symptomatic infection were higher at later time points after full vaccination (Odds Ratio [OR]_50 Days_: 2.22, 95% CI: 1.46 to 3.38; OR_100 Days_: 2.87, 95% CI: 2.01 to 4.11; OR_150 Days_: 3.36, 95% CI: 2.35 to 4.81; OR_200 Days_: 3.81, 95% CI: 2.67 to 5.44; and OR_250 Days_: 3.62, 95% CI: 2.52 to 5.20; Figure 2A and Table 1). Age, race, ethnicity, sex, and comorbidities were not strongly associated with the odds of symptomatic infection after full vaccination (Table S2, Supplementary Material). However, the odds of non-COVID-19 hospitalization or pneumonia (negative control outcomes) were significantly lower at later time points after BNT162b2 vaccination (Figure 2A and Table 1), suggesting possible sources of confounding in the design which could lead to underestimation of the degree of waning protection against symptomatic infection.

**Table 1.** Primary analysis: adjusted odds of symptomatic SARS-CoV-2 infection, non-COVID-19 hospitalization, and non-COVID-19 pneumonia by time since full BNT162b2 vaccination.

	Adjusted odds ratio (95% CI)		
Time relative to full vaccination date	Symptomatic infection [*N* = 5,985 events]	Non-COVID-19 hospitalization [*N* = 5,599 events]	Non-COVID-19 pneumonia [*N* = 447 events]
Day 0	1 (Reference)	1 (Reference)	1 (Reference)
Day 50	2.22 (1.46–3.38)	0.75 (0.63–0.9)	0.71 (0.36–1.41)
Day 100	2.87 (2.01–4.11)	0.52 (0.44–0.62)	0.35 (0.18–0.67)
Day 150	3.36 (2.35–4.81)	0.42 (0.35–0.5)	0.33 (0.16–0.69)
Day 200	3.81 (2.67–5.44)	0.31 (0.25–0.38)	0.33 (0.15–0.73)
Day 250	3.62 (2.52–5.2)	0.27 (0.21–0.35)	0.32 (0.12–0.85)

### Secondary analysis 1: change in the odds of infection over time after the first vaccine dose

Of 254,304 individuals who received at least one dose of BNT162b2 with no positive SARS-CoV-2 tests prior to vaccination, 42,529 underwent symptomatic testing after their first dose. There were 6,605 individuals who presented as positive cases (578 before and 6,027 after expected full vaccination, respectively) and 37,791 negative tests (3,084 before and 34,707 after expected full vaccination, respectively) from 31,330 individuals (Figure S1 and Table S3, Supplementary Material). Compared to 4 days after the first dose (a proxy for the unvaccinated state), the odds of symptomatic infection decreased through the expected second dose and full vaccination dates (e.g. OR_Day 21_: 0.62; 95% CI: 0.43 to 0.89 and OR_Day 35_: 0.16; 95% CI: 0.11 to 0.23), corresponding to the onset of VE (Figure 2B and Table 2). The odds at time points further removed from the first dose were higher than those at the expected full vaccination date but importantly remained lower than those at the proxy unvaccinated state (e.g. OR_Day 185_: 0.45, 95% CI: 0.34 to 0.61; OR_Day 185_: 0.45, 95% CI: 0.34 to 0.61; and OR_Day 235_: 0.52, 95% CI: 0.39 to 0.70; Figure 2B and Table 2).

**Table 2.** Secondary analysis: adjusted odds of symptomatic SARS-CoV-2 infection, non-COVID-19 hospitalization, and non-COVID-19 pneumonia by time since first BNT162b2 dose.

	Adjusted odds ratio (95% CI)		
Time relative to first vaccine dose	Symptomatic infection [*N* = 6,605 events]	Non-COVID-19 hospitalization [*N* = 6,441 events]	Non-COVID-19 pneumonia [*N* = 488 events]
Day 4	1 (Reference)	1 (Reference)	1 (Reference)
Days 10	1.23 (0.82–1.85)	0.93 (0.59–1.47)	0.24 (0.04–1.48)
Day 14	0.89 (0.63–1.25)	0.88 (0.61–1.27)	0.6 (0.16–2.25)
Day 21(Expected second dose)	0.62 (0.43–0.89)	0.86 (0.6–1.22)	0.23 (0.06–0.86)
Day 35(Expected full vaccination)	0.16 (0.11–0.23)	1 (0.72–1.38)	0.54 (0.18–1.59)
Day 85	0.26 (0.19–0.36)	0.78 (0.56–1.08)	0.43 (0.15–1.29)
Day 135	0.39 (0.29–0.53)	0.56 (0.4–0.78)	0.22 (0.07–0.68)
Day 185	0.45 (0.34–0.61)	0.46 (0.33–0.64)	0.22 (0.07–0.7)
Day 235	0.52 (0.39–0.7)	0.34 (0.24–0.48)	0.23 (0.07–0.72)
Day 285	0.5 (0.37–0.67)	0.32 (0.22–0.46)	0.22 (0.06–0.81)

### Secondary analysis 2: age subgroup analysis

Among individuals aged 18 to 44, 45 to 64, and 65+ years old, there were 2,419, 2,141, and 1,329 positive symptomatic tests (cases) after the first BNT162b2 dose, respectively. Subgroup analyses suggest that there are indeed trends of waning protection against symptomatic infection after full vaccination for all three groups (Figure S2 and Table S4, Supplementary Material). Importantly, in each group, the odds of symptomatic infection were still lower 285 days after the first dose compared to 4 days after the first dose, with odds ratios of 0.63 (95% CI: 0.38 to 1.05), 0.44 (95% CI: 0.25 to 0.76), and 0.26 (95% CI: 0.11 to 0.61) for the 18 to 44, 45 to 64, and 65 + groups, respectively (Figure S2 and Table S4, Supplementary Material).

### Sensitivity analysis: stratification on time of vaccination rather than time of testing

One source of confounding in our primary analysis, which could contribute to the unexpected negative control findings described previously, is that any given stratum may include individuals who became eligible or chose to get vaccinated at different times. To address this, we modified the CLR to instead stratify on the time of vaccination and the county-level COVID-19 incidence at the time of PCR testing (see Supplementary Material Methods). Among 219,399 individuals who received their first dose on or after 2020 December 15 and were at risk for infection at their date of full vaccination, there were subsequently 6,024 positive symptomatic tests (cases) and 39,703 negative symptomatic tests (controls) contributing to analyzable strata (Figure S1 and Table S5, Supplementary Material). With this modified approach, the odds of non-COVID-19 hospitalization and pneumonia (negative control outcomes) were less associated with time since BNT162b2 vaccination (Figure S3 and Table S6, Supplementary Material). There was a stronger signal for waning immunity than in the primary analysis, as the odds of symptomatic infection at 200 and 250 days after full vaccination were 18.0 (95% CI: 11.4 to 28.4) and 23.0 (95% CI: 14.4 to 36.8) times higher than at the date of full vaccination, respectively (Figure S3 and Table S6, Supplementary Material).

## Discussion

Taken together, these data show that the risk of symptomatic infection several months after BNT162b2 vaccination is higher than at the date of full vaccination but significantly lower than at a baseline or unvaccinated state. We were not adequately powered to assess the durability of protection against severe COVID-19 (e.g. hospitalization, ICU admission, and death) as these events were fortunately rare in all time periods for vaccinated individuals, but we acknowledge that these outcomes are of primary importance.

Importantly, these data do not indicate a complete loss of effectiveness against symptomatic infection over the duration of the study. Instead, the adjusted odds of experiencing a symptomatic infection remain lower even 250 days after expected full vaccination compared to 4 days after the first dose, when immunity more closely approximates the unvaccinated state (OR: 0.5, 95% CI: 0.37 to 0.67). This suggests that significant protection against symptomatic infection does persist for months after vaccination. That said, these findings are in agreement with recommendations to administer vaccine booster doses to individuals who received a primary two-dose series of BNT162b2 (27). Recent studies have highlighted the effectiveness and safety of boosters, and it is important to continue prospectively evaluating their effectiveness in preventing severe disease and against new variants (28–35).

It is noteworthy that other reports have suggested waning effectiveness of BNT162b2 against symptomatic infection over time (12–15, 20, 23, 36–40). The results presented here are consistent with these prior studies, but it should be noted that our data cannot be directly extrapolated to suggest that the same trends apply for other COVID-19 vaccines. Indeed, other analyses have demonstrated that effectiveness and durability are higher for mRNA-1273 than BNT162b2 (24, 40–43), and preliminary data suggests that Ad26.CoV.S may elicit more durable protection than both mRNA vaccines against symptomatic infection and hospitalization (44). Regarding BNT162b2 specifically, several studies have demonstrated that antibody titers decline over time after full vaccination, which is particularly relevant because neutralizing antibody levels are suggested to be highly predictive of protection against SARS-CoV-2 infection (16). In one study, the levels of Spike protein antibodies declined by approximately two-fold between 21 and 41 days and 70+ days after the second dose of BNT162b2 (18). In a separate study of healthcare workers, the levels of neutralizing antibodies and antibodies that specifically recognize the Spike protein receptor binding domain significantly declined over several months after full vaccination (17).

The data presented here complement these prior studies in a number of ways. First, this is among the longest-term durability analyses for the primary two-dose BNT162b2 series. While much attention has now been shifted to the effectiveness and durability of booster doses, it is important to continue gathering long-term data on the durability of the primary series given that over 50% of the eligible population in the United States remains unboosted as of April 2022 (7). Second, the geographic regions from which this study population is derived (i.e. the Midwest, Florida, and Arizona) have not been covered in prior durability studies. Finally, here we introduced a new method (in Secondary Analysis 1) which enables the approximation of VE in a cohort that consists exclusively of vaccinated individuals. Given the lack of a centralized vaccination registry in the United States and the difficulties of ensuring high-fidelity syncing between state registries and electronic health record (EHR) databases, it is difficult to confidently characterize individuals as unvaccinated based solely on the lack of a recorded vaccination in the EHR. The inevitable misclassification of some vaccinated individuals as unvaccinated will almost certainly lead to underestimations of VE. On the other hand, in our secondary analysis, we establish the baseline odds of infection by considering a high confidence “pseudo-unvaccinated state” (i.e. the first week after the administration of the first vaccine dose), during which immunologic protection against infection is not yet expected to have developed.

In this context, it is important to highlight that these primary and secondary analyses are integrated with and complementary to each other. Indeed, the analyses are conducted using highly overlapping cohorts, with the exception that individuals who received a single vaccine dose or who tested positive between their first and second doses are eligible to contribute only to the secondary analysis. While the primary analysis directly addresses the main question that we aimed to answer (i.e. whether protection against symptomatic infection declines in the months after full vaccination), the secondary analysis provides important additional context by estimating the actual degree of residual protection at any time point after full vaccination. Perhaps more importantly, the results derived from these analyses are quite consistent with each other. The primary analysis indicated that the odds of symptomatic infection are 3.62 times higher 250 days after full vaccination compared to the date of full vaccination. In the secondary analysis, the odds of infection at the date of full vaccination and 250 days after full vaccination are 0.16 and 0.5 times the odds of infection during the pseudo-unvaccinated state, indicating increased odds by 3.13 times over this time period.

We note that the negative controls assessed in this study (non-COVID-19 hospitalization and non-COVID-19 pneumonia) are suboptimal, as there are likely multiple complex factors relating these outcomes to patterns in vaccination status. A previous study analyzing the intraseasonal waning effectiveness of influenza vaccination considered the laboratory-based diagnosis of respiratory syncytial virus (RSV) as a negative control outcome, which is reasonable given that it is another respiratory illness which may present with similar symptoms to influenza but should not be impacted by influenza vaccination status (45). Unfortunately, the diagnosis of RSV or influenza was not a feasible negative control in this study, largely because these infections were diagnosed at historically low rates throughout much of the COVID-19 pandemic (46–48). Further, RSV testing at our centers is performed primarily in young children, immunocompromised adults, or patients who are being tested for influenza. The systematic clinical and temporal differences in testing patterns for RSV and influenza compared to SARS-CoV-2 would limit their utility as negative controls in this study.

In this modified test-negative study design to assess vaccine durability, our primary variable of interest is Time Since Vaccination which itself is defined as the difference between the testing date and the vaccination date. This poses an analytic challenge, as only the date of testing or vaccination (but not both) can be used to match/stratify cases and controls in any regression model. In most test-negative studies, it is standard to stratify on the date of testing (in addition to geographical location). Doing so inherently controls for natural fluctuations in community transmission levels over time, allowing us to assume that matched patients presenting with symptoms are similarly likely to contribute a positive test. However, in a durability analysis, such stratification on the testing date necessitates the comparison of individuals who were vaccinated during different time periods. Accordingly, this analysis may be subject to confounding factors that relate to differences in features such as (i) the baseline health status and/or health consciousness of individuals who were vaccinated in early versus late phases of the rollout, (ii) differences in symptomaticity thresholds that drive SARS-CoV-2 testing between early and late vaccine recipients, or (iii) the dynamic nature of elective procedures and non-COVID-19 related healthcare during the pandemic. To intentionally control for these factors, we performed a sensitivity analysis in which the regression was stratified on the date of vaccination rather than the date of testing. A shortcoming of this approach is that it provides less robust control for community transmission risk at the time of testing and for differential VE against SARS-CoV-2 variants, and so we additionally stratified on the trailing 7-day county-level COVID-19 incidence rate and included the state-level dominant variant as a covariate in the regression. The results were indeed consistent with the conclusion of the primary analysis but suggested a considerably higher degree of waning. It is worth noting the possibility that this sensitivity analysis would overestimate the degree of waning because the latest cases (i.e. those with the most time between vaccination and testing) are disproportionately due to the Omicron variant, which is more transmissible, immunoevasive, and likely to cause breakthrough infections than prior variants (49–55). Thus, we believe that the true degree of waning protection against symptomatic infection lies between the point estimates derived from these two approaches.

This study has limitations. First, the demographic composition of the studied cohort is not representative of the United States or global population (e.g. over 90% White). Future investigation should test whether these results apply to more diverse and representative populations. Second, due to the rarity of hospitalization, ICU admission, and death in our vaccinated cohorts, we were not able to robustly assess whether protection against these severe outcomes changes over time. Further, the lack of clinical documentation associated with the vast majority of COVID-19 tests (particularly negative tests, i.e. controls) prevented us from analyzing whether cases and controls tended to present with different symptomatic profiles and/or severities, and whether these patterns changed over the course of the study period. Third, there are individual level SARS-CoV-2 exposure risk factors and nonpharmaceutical interventions that could not be accounted for in our regression analyses such as occupational risk (e.g. healthcare worker status) and adherence to masking and social distancing guidelines. Fourth, the use of a test-negative study design makes it difficult to assess the durability of protection against asymptomatic infection. While the vaccines are primarily intended to reduce symptomatic infection and severe disease, asymptomatic infections comprise a meaningful fraction of cases and can contribute to community transmission (56–58). Fifth, the test-negative design can be adversely impacted by the variable and sometimes low sensitivity of SARS-CoV-2 PCR tests, which will likely result in the misclassification of some cases as controls (59, 60). Sixth, because the infecting SARS-CoV-2 variant was not determined for the majority of cases in this study, we are not able to directly assess differences in the effectiveness and/or durability of BNT162b2 against the Alpha, Delta, and Omicron variants. Finally, if an individual tested positive with an antigen test in the home setting, they would not be captured in this analysis unless they subsequently presented to the clinic for confirmatory PCR testing.

BNT162b2 demonstrated strong protection against symptomatic and severe disease in clinical trials and the real world setting during early phases of the vaccine rollout internationally (3, 5, 8, 9, 61–64). However, it is important to evaluate the durability of this protection over time and in the context of a rapidly evolving landscape of SARS-CoV-2 variants. This study demonstrates that BNT162b2 strongly protects against symptomatic infection for at least 8 months after full vaccination, but the degree of protection wanes over this period. Going forward, it will be important to continually monitor the durability of protection against symptomatic infection and severe disease for both the primary series and booster doses of each authorized COVID-19 vaccine.

## Materials and Methods

### Study population

This is a retrospective study of individuals who were vaccinated with BNT162b2 between 2020 December 15 and 2022 January 27, and who subsequently underwent polymerase chain reaction (PCR) testing for suspected symptomatic SARS-CoV-2 infection at the Mayo Clinic. According to the CDC, full vaccination with BNT162b2 is defined as beginning 14 days after the second dose (65). This study was reviewed and deemed exempt by the Mayo Clinic institutional review board. Those who had specifically opted out of inclusion of electronic medical records in research were excluded. Inclusion and exclusion criteria were defined as follows.

Inclusion criteria:

1. Age greater than or equal to 18 years as of 2020 December 15.
2. Received two doses of BNT162b2 per-protocol, with the first dose administered on or after 2020 December 15. Per-protocol BNT162b2 vaccination was defined as two doses administered 18 to 28 days apart with no doses of other COVID-19 vaccines (i.e. mRNA-1273, Ad26.COV2.S) administered at any time before the second dose.
3. At least one clinical encounter at the Mayo Clinic in the 3 years preceding the study start date (i.e. between 2017 December 15 and 2020 December 15), per the EHR.

Exclusion criteria:

1. Any positive SARS-CoV-2 PCR test prior to the date of full vaccination.

The derivation of this study population is illustrated in Figure 1 and Figure S1 (Supplementary Material), and the demographic and clinical characteristics of the cohort along with the underlying fully vaccinated population is shown in Table S1 (Supplementary Material).

### Study design

We performed a test-negative case-control analysis to assess whether the protection conferred by BNT162b2 wanes over time, similar to a study design described previously to analyze intraseasonal waning effectiveness of influenza vaccination (45). To do so, we used conditional logistic regression (CLR) to assess the odds of symptomatic SARS-CoV-2 infection and two negative control outcomes (non-COVID-19 hospitalization and non-COVID-19 pneumonia) over time after full vaccination, while adjusting for relevant covariates. Because we expect the date of full vaccination to approximate the time of maximal protection, we assess vaccine durability by estimating the odds of symptomatic infection at 50, 100, 150, 200, and 250 days after this time point.

Symptomatic infection was defined as a positive result from a SARS-CoV-2 PCR test that was not designated as “asymptomatic” by the ordering provider (subsequently referred to as “symptomatic tests”). There are not strict criteria for the designation of a test as symptomatic versus asymptomatic, but guidelines are derived from the Mayo Clinic COVID-19 Navigator (66). The following signs and symptoms are currently considered consistent with possible SARS-CoV-2 infection in adults: fever, fatigue, dry cough, anosmia, dyspnea, myalgia, chills, shaking with chills, headache, diarrhea, nausea or vomiting, chest pain, rhinorrhea, conjunctivitis, sore throat, and rash.

### Definitions of cases, controls, and at-risk time

Cases were defined as the first positive symptomatic test for a given individual; if an individual contributed multiple positive tests, only their first test was included as a case. Controls were defined as negative symptomatic tests which did not occur after any prior positive SARS-CoV-2 PCR tests (asymptomatic or symptomatic). Individuals who met the inclusion and exclusion criteria outlined above were eligible to contribute cases and controls from their date of full vaccination until they (i) had any positive test result (symptomatic or asymptomatic), (ii) received a third dose of any COVID-19 vaccine (BNT162b2, mRNA-1273, or Ad26.COV2.S), (iii) died, or (iv) reached the end of the study period. If an individual contributed a negative symptomatic test 15 or fewer days before a positive test, that negative test was excluded as a possible false negative. If an individual contributed multiple negative symptomatic tests within 15 days of each other, then one of those tests was randomly selected as a control while the others were dropped; this step was taken to avoid counting multiple controls from a potential single symptomatic illness. Further, if an individual contributed more than three negative symptomatic tests over the study duration, then three tests were randomly selected as controls while the others were dropped, as was recently described in a test-negative case-control study of COVID-19 VE (67).

As a negative control analysis, we assessed protection against non-COVID-19 hospitalization and non-COVID-19 pneumonia, outcomes which we do not expect to be impacted by time since vaccination. In other test negative designs on influenza, other respiratory infections were used as the negative control (45). Because of the myriad impacts of the pandemic and nonpharmaceutical interventions on other respiratory infections (e.g. very low rates of influenza in 2020 and through most of 2021), such an approach may not be valid here (46). Although non-COVID-19 related hospitalization could be impacted by factors such as changes in healthcare-seeking behavior (including elective procedures) after vaccination, it appeared to be a reasonable negative control to evaluate. For non-COVID-19 hospitalization, cases were defined as instances in which an individual experienced a negative symptomatic test (i.e. ruled out for COVID-19 diagnosis) and was subsequently admitted to the hospital within 14 days. Controls were defined as instances in which an individual experienced a negative symptomatic test and was not subsequently admitted to the hospital within 14 days. Individuals who met the inclusion and exclusion criteria outlined above were eligible to contribute cases and controls from their date of full vaccination until they (i) were hospitalized within 14 days of a negative symptomatic test, (ii) received a third dose of any COVID-19 vaccine (BNT162b2, mRNA-1273, or Ad26.COV2.S), (iii) died, or (iv) reached the end of the study period. The same rules were applied as described above for cases in which an individual contributed (i) a negative test shortly before contributing a positive test, (ii) multiple negative symptomatic tests within 15 days of each other, or (iii) more than three negative symptomatic tests over the duration of the study. Because 14 days of follow-up were required after a positive symptomatic test to observe this outcome, cases and controls were only considered from tests that were performed on or before 2022 January 17 (14 days before the last date of data collection). A similar process was followed for non-COVID-19 pneumonia, except that cases were defined by the presence of at least one corresponding ICD-10 code (J12–J18, with the exceptions of J12.81, J12.82, and J12.89) within 14 days of a negative symptomatic test.

### Primary exposure, covariates, and stratification factors

Variables that are potentially associated with the likelihood of eligibility for vaccination at a given time, seeking out vaccination, testing positive for SARS-CoV-2, or experiencing severe COVID-19 were included as covariates or stratification variables in the regression models. The primary exposure of interest and each such other variable, denoted as X_1_–X_15_ in the regression equation listed in the *Statistical Analysis* section, is described below.

Primary exposure:

1. X_1_: time since full vaccination, defined as the number of days between the symptomatic PCR test and the date of full vaccination. This variable was modeled as a linear spline with knots at 50-day intervals since the full vaccination date. As described above, the date of full vaccination is expected to correspond to a time of maximal protection, and thus was considered as the reference. Results are presented as the odds of symptomatic infection at each knot relative to this reference.

Covariates:

1. X_2_: age in years as of the study start date (2020 December 15), modeled as a linear spline with knots at 25, 35, 45, 55, 65, 75, and 85 years. The minimum age (18 years old) was considered the reference. Results are presented as the odds of symptomatic infection at each subsequent knot relative to this reference.
2. X_3_–X_10_: individual comorbidity categories, binarized based on whether the individual had at least one instance of a corresponding ICD-10 code in the 5 years prior to the study period. The comorbidity categories included cardiovascular disease, pulmonary disease, diabetes, kidney disease, liver disease, HIV/AIDS, cancer, and obesity. The mapping of ICD codes to comorbidities is described in further detail below.
3. X_11_: race, categorized into seven groups (listed alphabetically: Asian, Black/African American, Native American, Native Hawaiian/Pacific Islander, other, White, and unknown). White was considered the reference category because it comprised the majority of individuals in the study.
4. X_12_: ethnicity, categorized into three groups (listed alphabetically: Hispanic/Latino, not Hispanic/Latino, and unknown). “Not Hispanic/Latino” was considered the reference category because it comprised the majority of individuals in the study.
5. X_13_: sex, categorized into three groups (listed alphabetically: female, male, and unknown). Female was considered the reference category.

Stratification factors:

1. X_14_: county of residence at the time of testing for the individual who underwent the symptomatic test.
2. X_15_: calendar time of test, categorized in 1-week intervals starting on the date of the first symptomatic test after full vaccination.

### Determination of comorbidities

We used the *comorbidity* package (version 0.5.3) in R (version 4.1.0, www.r-project.org, Vienna, Austria) to identify ICD-9 and ICD-10 codes that correspond to the comorbidity categories listed above. For each individual, we extracted all such diagnosis codes in the Mayo Clinic EHR from the 5 years preceding this study (i.e. between 2015 December 15 and 2020 December 15). The eight comorbidity categories were defined as one or more diseases from the Elixhauser score as follows:

1. Cardiovascular disease: congestive heart failure (chf), cardiac arrhythmias (carit), valvular disease (valv), pulmonary circulation disorders (pcd), peripheral vascular disorders (pvd), uncomplicated hypertension (hypunc), and complicated hypertension (hypc).
2. Pulmonary disease: chronic pulmonary disease (cpd).
3. Diabetes: uncomplicated diabetes (diabunc) and complicated diabetes (diabc).
4. Kidney disease: renal failure (rf).
5. Liver disease: liver disease (lf).
6. HIV/AIDS: AIDS/HIV (aids).
7. Cancer: lymphoma (lymph), metastatic cancer (metacanc) and solid tumor without metastasis (solidtum).
8. Obesity: obesity (obes).

### Statistical analysis

Briefly, for each outcome (i.e. symptomatic SARS-CoV-2 infection, non-COVID-19 hospitalization, and non-COVID-19 pneumonia), we fit a CLR model to estimate the odds of experiencing the outcome of interest each day after the date of full vaccination compared to the odds of experiencing that outcome on the date of full vaccination, while adjusting for the covariates described above.

The CLR models were each defined by the equation

$log\left( {\frac{{{p_{Outcome}}}}{{1\,\, - \,\,{p_{Outcome}}}}} \right)\,\, = {\beta _0} + {\beta _1}{X_1} + {\beta _2}{X_2} + \ldots + {\beta _{13}}{X_{13}} + Strata\left[ {{X_{14}}, {X_{15}}} \right],$

where the covariates and stratification variables X_1_–X_15_ are described in the section above.

Models were fit using the *clogit* function from the *survival* package (version 3.2.11) in R (Version, 4.1.0, www.r-project.org, Vienna, Austria). CI and tests were based upon the Wald method, and the Efron method was used to approximate the conditional likelihood. Odds ratios were considered statistically significant if the CI did not include 1. In addition, Nagelkerke *R*-squared values are reported for each model in Table S7 (Supplementary Material).

### Multicollinearity analysis

To assess multicollinearity among the covariates for each CLR model, we computed the Variance Inflation Factor (VIF) for each of the nonstrata covariates in each model. These values are provided in Table S8 (Supplementary Material). A cutoff VIF threshold of 5 was used to identify covariates which are significantly collinear with respect to the other model covariates. VIF values were computed using the *mctest* package (version 1.3.1) in R.

#### Secondary and sensitivity analyses

We performed secondary analyses to (i) estimate VE as a function of time (rather than only assessing whether the odds of infection increases after the date of full vaccination) and (ii) assess the applicability of our findings on individuals in different age subgroups. We also performed a sensitivity analysis to address possible confounding factors in the primary analysis due to the stratification on testing date. In this sensitivity analysis, cases and controls were instead stratified on the date of vaccination, and additional covariates (e.g. SARS-CoV-2 variant prevalence and regional COVID-19 incidence at the time of PCR testing) were included in the regression models (68, 69). These additional analyses are described in further detail in the Supplemental Material.

## Supplementary Material

pgac082_Supplemental_File

## Data Availability

The datasets supporting the current study have not been deposited because they contain personally identifiable information from human subjects. This data may be made available from the corresponding author on request (Venky Soundararajan; venky@nference.net). A proposal with a detailed description of the study objectives and statistical analysis plan will be needed to evaluate the reasonability of requests. Deidentified data will be provided after approval from the lead contact and the Mayo Clinic's standard IRB process for such requests.

## Notes

**Competing Interest:** A.P., P.J.L., C.P., M.J.N., A.V., and V.S. are employees of nference. J.C.O. has received small grants from nference, Inc., and personal consulting fees from Bates College and Elsevier Inc. All of these activities are outside of the present work. M.D.S. received research funding for the HEROES Together vaccine SE registry from Pfizer via Duke University. A.V. reports being an inventor for the Mayo Clinic Travel App interaction with the Smart Medical Kit and the Medical Kit for Pilgrims. A.D.B. is a paid consultant for Abbvie, Gilead, Freedom Tunnel, Pinetree therapeutics Primmune, Immunome, MarPam, and Flambeau Diagnostics, is a paid member of the DSMB for Corvus Pharmaceuticals, Equilium, and Excision Biotherapeutics, has received fees for speaking for Reach MD and Medscape, owns equity for scientific advisory work in Zentalis and nference, and is founder and President of Splissen therapeutics. W.K., J.E.G., H.L.G., and L.L.S. have no interests to disclose. The Mayo Clinic holds equity in the company sponsoring the analysis presented in this manuscript.
