# Supplementary material for: Durability analysis of the highly effective BNT162b2 vaccine against COVID-19
Source: PNAS Nexus. 2022 Jun 8;1(3):pgac082. doi: 10.1093/pnasnexus/pgac082 (PMC9272171; doi:10.1093/pnasnexus/pgac082)
Supplement: pgac082_Supplemental_File [file pgac082_supplemental_file.docx]

# **Supplemental Information**

# **Supplemental Methods**

***Secondary Analysis 1: odds of infection relative to the first vaccine dose rather than full vaccination***

The primary analysis estimates the change in odds of infection over time relative to the time of maximal vaccine protection without providing the important context of the baseline risk of infection in the absence of vaccination. In our study population, which consists entirely of vaccinated individuals with no prior history of COVID-19 diagnosis, it is not possible to directly measure this risk of infection in the unvaccinated state. However, the protective effect of BNT162b2 sets in after approximately two weeks, and we reasoned that the risk of infection in the unvaccinated state could be approximated by the risk of infection shortly after initial vaccination (e.g., within days 4-10 after the first dose).[^3,9^](https://paperpile.com/c/nLavOW/Xh9wx+bWCVj) We thus modified the inclusion and exclusion criteria criteria from our primary analysis as follows.

Inclusion criteria:

1. Age greater than or equal to 18 years as of December 15, 2020.
2. Received at least one dose of BNT162b2 on or after December 15, 2020.
3. At least one clinical encounter at the Mayo Clinic in the three years preceding the study start date (i.e. between December 15, 2017 and December 15, 2020), per the electronic health record.

Exclusion criteria:

1. Any positive SARS-CoV-2 PCR test prior to the date of full vaccination.
2. Received one or more doses of another COVID-19 vaccine (mRNA-1273 or Ad26.COV2.S) on or before December 15, 2020.

Cases and controls were defined as described above but with modifications to the censoring protocol. Specifically, individuals who met the inclusion and exclusion criteria were eligible to contribute cases and controls from four days after their first vaccine dose until they (i) had any positive test result (symptomatic or asymptomatic), (ii) went off-protocol for their vaccination regimen (i.e. received a second dose of BNT162b2 less than 18 days after the first dose, did not receive a second dose of BNT162b2 by 28 days after their first dose, or received a dose of a different COVID-19 vaccine within 28 days of their first dose), (iii) received a third dose of any COVID-19 vaccine, (iv) died, or (v) reached the end of the study period. Note that at-risk time was defined to begin four days after the first dose (rather than the first day after the first dose) for two reasons: (i) individuals with respiratory symptoms were often encouraged to delay vaccination, resulting in a potential bias toward lower symptomatic infection rates immediately following vaccination; and (ii) individuals who develop symptomatic COVID-19 shortly after vaccination may attribute their symptoms to vaccine side effects, resulting in a likely delay of testing.

The same CLR model described above for the main analysis was applied, except that the “Time since vaccination” variable was now modeled as a linear spline with the following knots: 10, 14, 21, 35, 85, 135, 185, 235, and 285 days after the first dose. It is recommended that the second dose of BNT162b2 is administered 21 days after the first, with full vaccination thus expected to start 35 days after the first dose. Because the vaccine is not expected to provide protection until about two weeks after the first dose, we considered 4 days after the first dose as a reference time point to approximate unvaccinated status.[^3,5,9^](https://paperpile.com/c/nLavOW/Xh9wx+W7KP9+bWCVj) Results are presented as the odds of symptomatic infection at each knot relative to this reference.

***Secondary Analysis 2: Age subgroup analysis relative to the first vaccine dose.***

While the previous approaches included age as a covariate, we considered it important to determine whether any observed signal of waning was observed across all age groups. We thus divided the cohort of individuals vaccinated with one or two doses of BNT162b2 (i.e. the cohort from Secondary Analysis 1) into three age groups (based on age as of December 15, 2020): 18-44, 45-64, and ≥65 years. For each age group, we then fit the same CLR model as was described above for the secondary analysis (still including age as a covariate) to evaluate the association between time since first vaccination (i.e. four days after the first dose) and the odds of symptomatic infection or non-COVID-19 hospitalization.

***Sensitivity Analysis: Stratification by time of vaccination rather than by time of testing.***

By performing CLR with stratification by the time of testing, individuals with more time since vaccination in any given stratum will inherently have been vaccinated at earlier times during the vaccine rollout. Because the time of vaccination is likely associated with the risk of SARS-CoV-2 infection and the likelihood of engagement with the healthcare system, this approach could yield biased estimates of the infection odds over time after vaccination. We thus performed a sensitivity analysis in which the CLR was stratified by county, date of full vaccination (in two-week calendar intervals), and county-level COVID-19 incidence at the time of testing, rather than on county and time of testing (as was the case in the previous model). Here, we also included an additional covariate to capture the dominant SARS-CoV-2 variant at the time of the test; this was not included in our primary model because the variant prevalence was implicitly captured by stratifying on the time of testing. Specifically, the additional variables considered here were:

Covariates:

1. X_16_: Dominant SARS-CoV-2 variant, categorized as Alpha, Delta, Omicron, Neither, or Unknown. The prevalence of Alpha, Delta, and Omicron variants was determined for each state in twice-monthly intervals (i.e. from the first to the 15th day of each month, and from the 16th to the last day of each month) using publicly deposited whole genome sequences in the National Center for Biotechnology (NCBI) database.[^69^](https://paperpile.com/c/nLavOW/zPV5) For a given test, which is characterized by a specific combination of state and twice-monthly interval, this variable was denoted as (i) Alpha if the prevalence of Alpha variant sequences was > 0.5, (ii) Delta if the prevalence of Delta variant sequences was > 0.5, (iii) Omicron if the prevalence of Omicron variant sequences was > 0.5, (iv) neither if the prevalence of each of these variants was < 0.5, or (v) Unknown if there were fewer than 50 sequences deposited.
2. X_17_: Community level exposure risk, modeled as a linear spline with knots at 10, 25, 50, 75, 100, and 150 cases per 100,000 individuals. For a given county on a given day of testing, community exposure risk was proxied by the trailing 7-day average COVID-19 incidence.[^70^](https://paperpile.com/c/nLavOW/C11Qr)
3. X_18_: Community level exposure risk, categorized into seven buckets based on the same values used for the spline knots in variable X_17_.
4. X_19_: Calendar time of full vaccination for the individual who underwent the symptomatic test, categorized in two-week intervals.

The CLR model was then defined by the equation,

$log(\frac{p{}_{Outcome}}{1 - p{}_{Outcome}}) =$ β_0_ + β_1_X_1_ + β_2_X_2_ + … + β_13_X_13_ + β_16_X_16_ + β_17_X_17_ + Strata[X_14_, X_18_, X_19_], where the covariates and conditioning variables X_1_-X_13_ are the same as described above for the primary analysis. This equation mirrors that used in the primary analysis, except that X_16_ (dominant SARS-CoV-2 variant) and X_17_ (splined community level exposure risk) were added as covariates, and the stratifying variable X_15_ (calendar time of test) was replaced with X_18_ (bucketed community level exposure risk) and X_19_ (calendar time of vaccination).

# **Supplemental Figures**

**
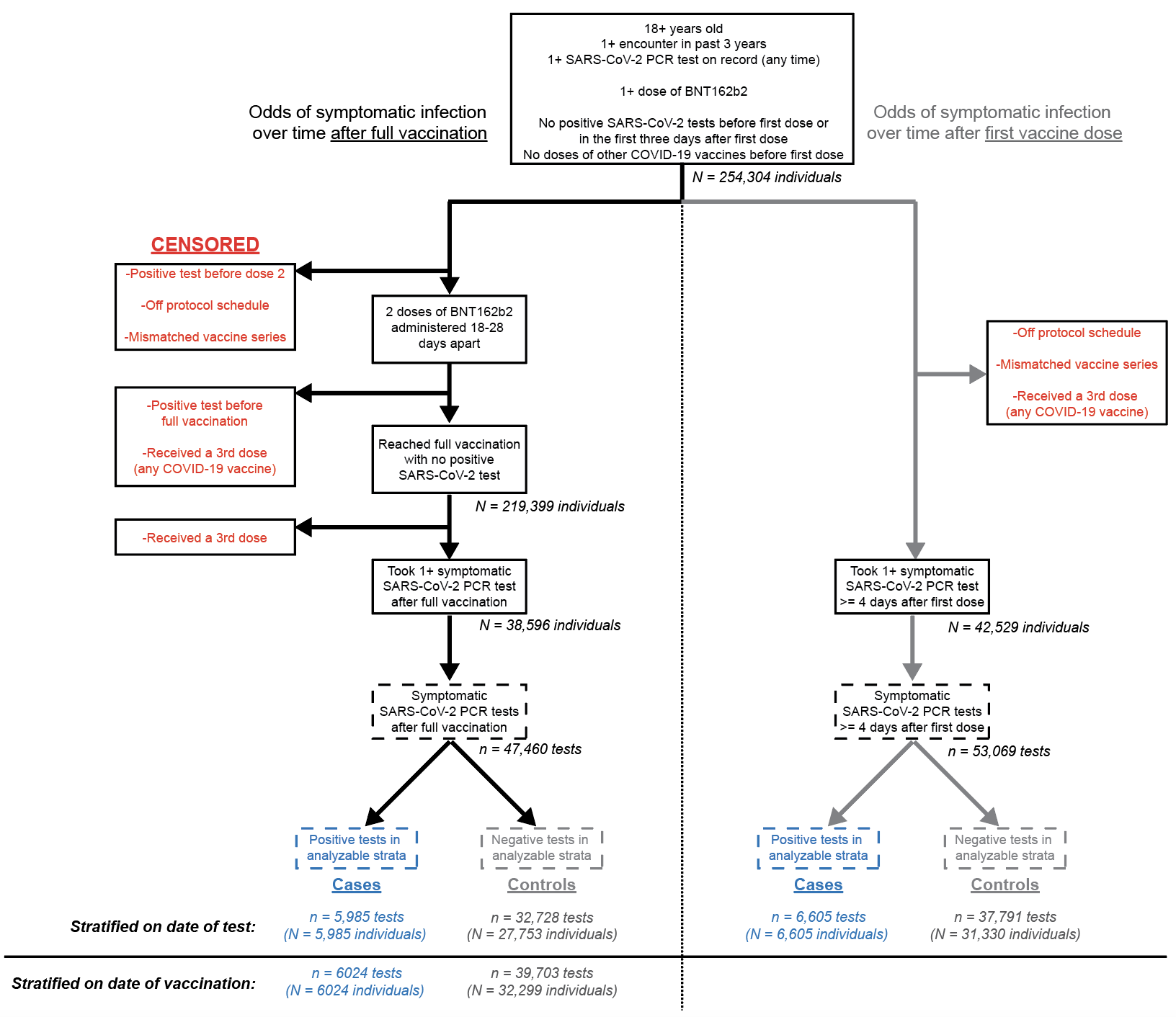
**

**Figure S1. Cohort development flowchart showing derivation of the various cohorts analyzed in this study.** Cohort derivation is depicted for three different study designs that were considered. On the left, the flow chart depicts the cohort derivation to analyze the odds of symptomatic infection after full vaccination, stratified by either the date of testing (Primary Analysis) or the date of vaccination (Secondary Analysis 2). On the right, the flow chart depicts the cohort derivation to analyze the odds of symptomatic infection after the first dose, stratified by the date of testing (Secondary Analysis 1). For the conditional logistic regression, a “stratum” is defined either as a unique combination of county (geography) and calendar time of testing, or a unique combination of county, calendar time of vaccination, and county-level COVID-19 incidence at the time of testing. An “analyzable stratum” is any such stratum which contains at least one case and at least one control. An individual was considered to have gone “off protocol schedule” if they (i) received a second dose of BNT162b2 less than 18 days after the first dose, (ii) did not receive a second dose of BNT162b2 by 28 days after the first dose, or (iii) received a dose of a different COVID-19 vaccine within 28 days of the first BNT162b2 dose. Boxes with solid outlines correspond to counts of individuals, and boxes with dotted outlines correspond to counts of tests. In the test-negative analyses, cases and controls were defined at the level of symptomatic tests. One individual is allowed to contribute multiple negative symptomatic tests, provided that those tests were separated from each other by at least 15 days. If an individual experienced any negative symptomatic tests prior to a positive test, those negative tests can contribute to the control set and the positive test can contribute to the case set.

**
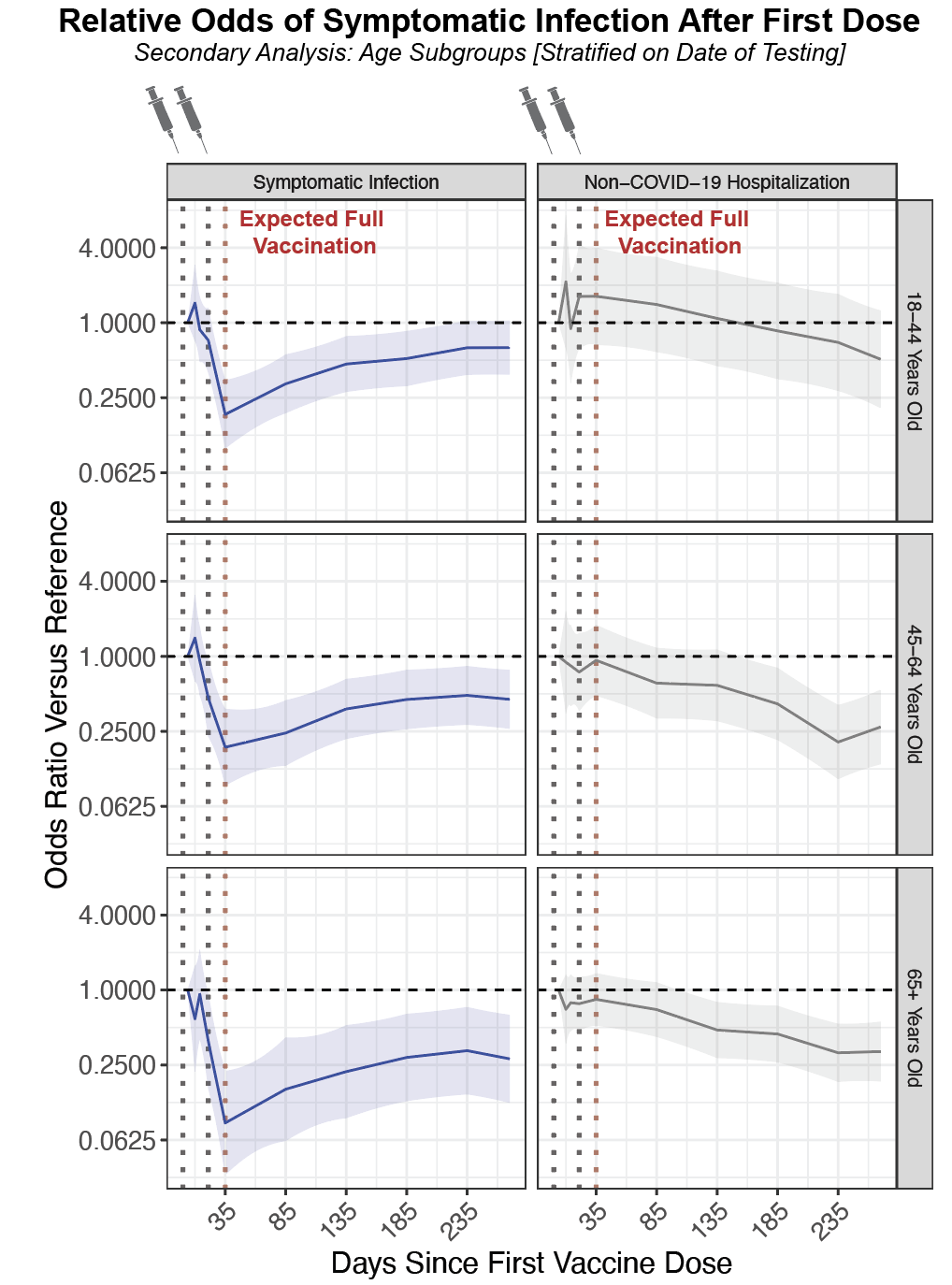
**

**Figure S2. Secondary analysis 2: relationship between time since first vaccine dose and the adjusted odds of outcomes of interest in three age subgroups.** The outcomes are symptomatic SARS-CoV-2 infection (blue; n = 2419 for 18-44; n = 2141 for 45-64; n = 1329 for 65+) and non-COVID-19 hospitalization (gray; n = 791 for 18-44; n = 1306 for 45-64; n = 3472 for 65+). Each curve indicates the adjusted odds ratio comparing the odds of experiencing the outcome at the given time compared to four days after the first dose, which is expected to approximate the unvaccinated state. The adjusted odds ratios at each day are calculated from the linear spline equations, and data is shown through the 90th percentile of the “Days Since First Dose” for the entire cohort. Data is shown for individuals 18-44 (top), 45-64 (middle), and at least 65 (bottom) years old. The shaded region indicates the 95% confidence interval of the odds ratio.

**
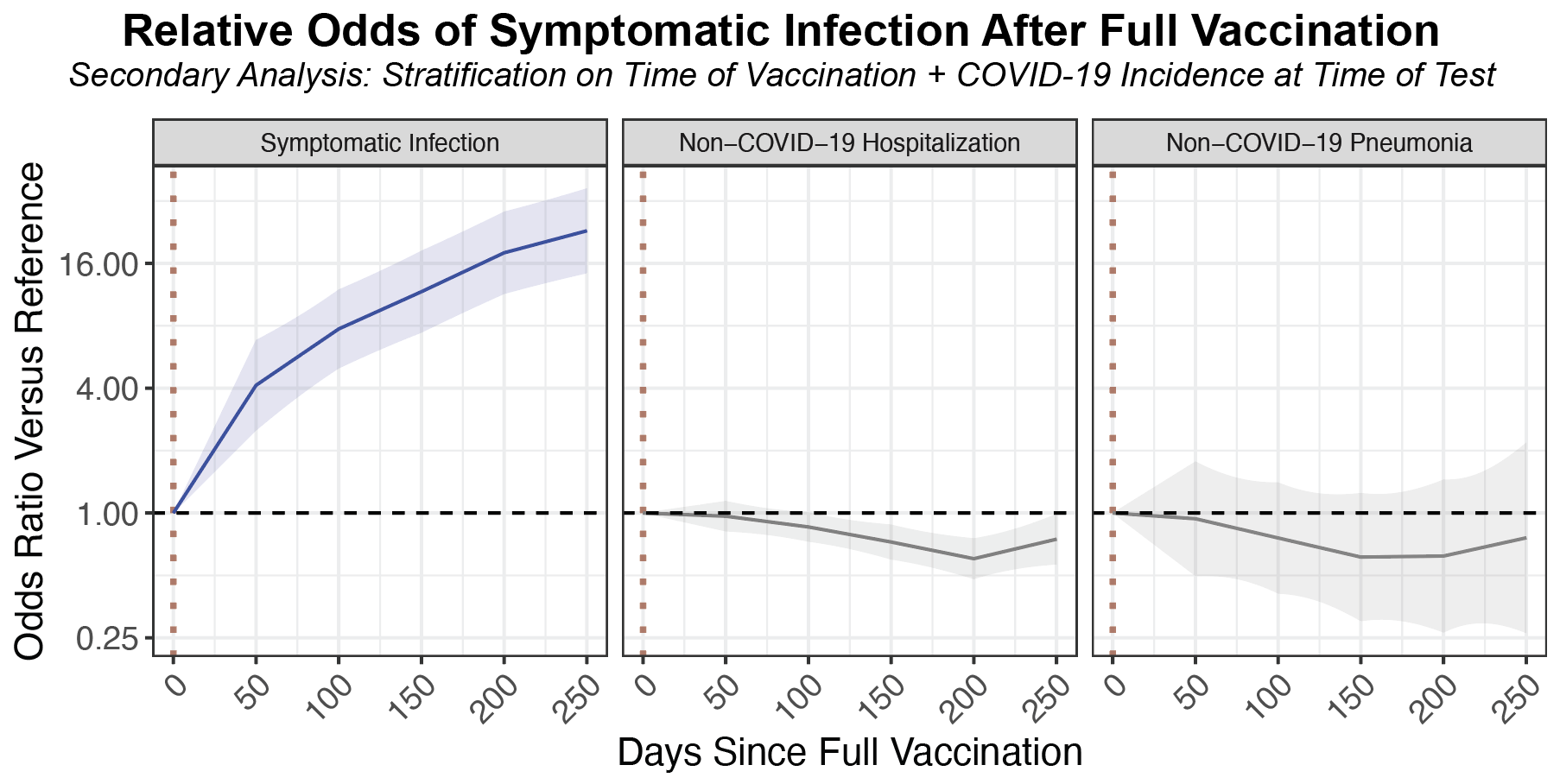
**

**Figure S3. Sensitivity analysis: relationship between time since full vaccination and adjusted odds of outcomes of interest with matching on time of vaccination and county-level COVID-19 incidence.** The outcomes are symptomatic SARS-CoV-2 infection (blue; 6024 cases), non-COVID-19 hospitalization (gray in middle; 5728 cases), and non-COVID-19 pneumonia (gray on right; 454 cases). Each curve indicates the adjusted odds ratio comparing the odds of experiencing the outcome at the given time compared to at the time of full vaccination, which is expected to correspond to maximal vaccine-mediated protection. The adjusted odds ratios at each day are calculated from the linear spline equations, and data is shown through the 90th percentile of the “Days Since Full Vaccination” for the entire cohort. The shaded region indicates the 95% confidence interval of the odds ratio.

# **Supplemental Tables**

**Table S1. Demographic and clinical characteristics of cases and controls for primary analysis of symptomatic infection among fully vaccinated BNT162b2 recipients.** The underlying population corresponds to the set of individuals who received their first BNT162b2 dose on or after December 15, 2020 and were fully vaccinated per protocol (i.e. with two doses administered 18-28 days apart and with no prior positive SARS-CoV-2 PCR tests before the date of full vaccination). The eligible population corresponds to the subset of the underlying population who underwent at least one symptomatic test after the date of full vaccination. Cases correspond to the first positive symptomatic test for a given individual in the eligible population; by definition, the number of individuals contributing cases is the same as the number of cases. Controls correspond to negative symptomatic tests after full vaccination which occur before the given individual has experienced any positive SARS-CoV-2 PCR tests; an individual can contribute multiple controls during the study period, so the number of individuals in the control population is less than the total number of tests (controls) contributed. Because an individual can contribute negative tests (controls) prior to contributing a positive test, the number of individuals in the eligible population is smaller than the sum of the number of individuals in the case and control populations. Sub-sampling in the control population refers to the process in which negative tests from a given individual were (i) excluded if they occurred after a positive test or within the 15 days before a positive test (possible false negative), (ii) randomly sampled if they occurred within 15 days of each other (possibly during the same symptomatic illness), and (iii) randomly sampled if the individual contributed more than three negative tests during the study period. A stratum (defined by the regression equation as a unique combination of county and calendar week of testing) is considered analyzable if it includes at least one case and at least one control, because strata including only cases or only controls do not contribute to the estimation of the regression coefficients. For all cases and controls, all summarized characteristics correspond to only individuals who contributed at least one symptomatic test to an analyzable stratum.

| **Characteristic** | **Underlying Population**    Fully vaccinated per-protocol | **Eligible Population**  Fully vaccinated per-protocol with subsequent symptomatic test | **Case Population**  1+ positive symptomatic test after full vaccination | **Control Population**  1+ negative symptomatic test after full vaccination, sub-sampled |
| --- | --- | --- | --- | --- |
| Number of individuals | 219399 | 38596 | 6081 | 33771 |
| Number of symptomatic tests |  | 47460 | 6081 | 41379 |
| Number of symptomatic tests in analyzable strata  *Total*  *Bucketed by time since full vaccination date*   - Days 0-29 - Days 30-59 - Days 60-89 - Days 90-119 - Days 120-149 - Days 150-179 - Days 180-209 - Days 210-239 - Days 240-269 - Days 270-299 - Days 300-329 - Days 330-359 |  |  | 5985  77  132  273  488  687  1063  1331  997  488  303  128  18 | 32728  1638  1734  2646  3695  4744  5779  5811  3893  1678  776  300  34 |
| Number of individuals contributing symptomatic tests in analyzable strata |  |  | 5985 | 27753 |
| Age (years)   - Mean (sd) - 18-24 - 25-34 - 35-44 - 45-54 - 55-64 - 65-74 - 75-84 - 85+ | 51.1 (21.0)  13,319 (6.1%)  24,890 (11.3%)  27,570 (12.6%)  29,121 (13.3%)  39,752 (18.1%)  35,737 (16.3%)  23,066 (10.5%)  6,910 (3.1%) | 52.0 (18.8)  2,613 (6.8%)  6,227 (16.1%)  6,714 (17.4%)  5,629 (14.6%)  7,069 (18.3%)  5,137 (13.3%)  3,683 (9.5%)  1,524 (3.9%) | 50.8 (17.7)  353 (5.9%)  949 (15.9%)  1,213 (20.3%)  992 (16.6%)  1,149 (19.2%)  685 (11.4%)  475 (7.9%)  169 (2.8%) | 50.7 (18.6)  2,059 (7.4%)  4,873 (17.6%)  4,969 (17.9%)  4,121 (14.8%)  5,057 (18.2%)  3,415 (12.3%)  2,325 (8.4%)  934 (3.4%) |
| State of Primary Residence   - Arizona - Florida - Iowa - Minnesota - Wisconsin - Other | 24,294 (11.1%)  27,658 (12.6%)  2,806 (1.3%)  111,664 (50.9%)  42,828 (19.5%)  10,149 (4.6%) | 2,573 (6.7%)  4,062 (10.5%)  244 (0.6%)  20,280 (52.5%)  11,331 (29.4%)  106 (0.3%) | 374 (6.2%)  544 (9.1%)  32 (0.5%)  3,148 (52.6%)  1,887 (31.5%)  0 (0.0%) | 1,837 (6.6%)  3,045 (11.0%)  38 (0.1%)  14,671 (52.9%)  8,162 (29.4%)  0 (0.0%) |
| Sex   - Female - Male - Unknown | 123,160 (56.1%)  96,215 (43.9%)  23 (0.0%) | 24,162 (62.6%)  14,434 (37.4%)  0 (0.0%) | 3,583 (59.9%)  2,402 (40.1%)  0 (0.0%) | 17,594 (63.4%)  10,159 (36.6%)  0 (0.0%) |
| Race   - Asian - Black/African American - Native American - Hawaiian/Pacific Islander - White - Other - Unknown | 7,557 (3.4%)  6,686 (3.0%)  659 (0.3%)  241 (0.1%)  192,808 (87.9%)  5,221 (2.4%)  6,227 (2.8%) | 1,211 (3.1%)  931 (2.4%)  110 (0.3%)  34 (0.1%)  35,301 (91.5%)  746 (1.9%)  263 (0.7%) | 154 (2.6%)  128 (2.1%)  18 (0.3%)  9 (0.2%)  5,537 (92.5%)  100 (1.7%)  39 (0.7%) | 942 (3.4%)  732 (2.6%)  82 (0.3%)  23 (0.1%)  25,205 (90.8%)  571 (2.1%)  198 (0.7%) |
| Ethnicity   - Hispanic or Latino - Not Hispanic or Latino - Unknown | 9,086 (4.1%)  201,144 (91.7%)  9,169 (4.2%) | 1,391 (3.6%)  36,681 (95.0%)  524 (1.4%) | 198 (3.3%)  5,712 (95.4%)  75 (1.3%) | 1,041 (3.8%)  26,317 (94.8%)  395 (1.4%) |
| Comorbidities   - Cancer - Cardiovascular disease - Diabetes - HIV/AIDS - Kidney disease - Liver disease - Obesity - Pulmonary disease | 9,797 (5.2%)  35,088 (18.8%)  10,309 (5.5%)  103 (0.1%)  9,084 (4.9%)  5,905 (3.2%)  18,497 (9.9%)  14,508 (7.8%) | 1,912 (5.2%)  8,336 (22.6%)  2,665 (7.2%)  21 (0.1%)  2,244 (6.1%)  1,357 (3.7%)  4,991 (13.6%)  3,941 (10.7%) | 215 (3.7%)  1,139 (19.9%)  387 (6.7%)  5 (0.1%)  288 (5.0%)  169 (2.9%)  754 (13.1%)  516 (9.0%) | 1,235 (4.7%)  5,656 (21.4%)  1,755 (6.7%)  15 (0.1%)  1,423 (5.4%)  941 (3.6%)  3,424 (13.0%)  2,770 (10.5%) |
| Dates of full vaccination   - Earliest - 25th % - Median - 75th % - Latest | 1/4/21  3/5/21  4/14/21  5/17/21  1/31/22 | 1/18/21  2/25/21  4/5/21  5/5/21  12/28/21 | 1/20/21  3/1/21  4/12/21  5/10/21  12/28/21 | 1/18/21  2/25/21  4/7/21  5/6/21  12/28/21 |
| Time between full vaccination and test   - Minimum - 25th % - Median - 75th % - Maximum |  | 0  87  149  197  343 | 1  143  186  221  343 | 0  108  160  201  340 |

**Table S2. Primary analysis: adjusted odds of symptomatic SARS-CoV-2 infection, non-COVID-19 hospitalization, and non-COVID-19 pneumonia for all non-strata covariates other than the primary exposure variable (time since full vaccination).** In total, there were 5985 positive symptomatic tests, 5599 non-COVID-19 hospitalizations, and 447 cases of non-COVID-19 pneumonia which contributed to analyzable strata. Adjusted odds were estimated with linear spline equations for age or by exponentiating the coefficients derived from conditional logistic regression models fit separately for each outcome. For “Time Relative to Full Vaccination”, knots through the 90th percentile of the days since full vaccination for the entire cohort are included.

|  |  | **Adjusted Odds Ratio (95% CI)** | | |
| --- | --- | --- | --- | --- |
| **Covariate** | **Level / Category** | **Symptomatic Infection**  **[N = 5985 events]** | **Non-COVID-19 Hospitalization**  **[N = 5599 events]** | **Non-COVID-19 Pneumonia**  **[N = 447 events]** |
| Age | 18 | 1 (Reference) | 1 (Reference) | 1 (Reference) |
|  | 25 | 1.17 (0.9-1.5) | 1.3 (0.88-1.92) | 0.59 (0.09-3.77) |
|  | 35 | 1.43 (1.16-1.76) | 1.01 (0.73-1.41) | 0.31 (0.07-1.47) |
|  | 45 | 1.73 (1.39-2.15) | 1.18 (0.84-1.67) | 0.85 (0.19-3.82) |
|  | 55 | 1.63 (1.31-2.02) | 2.14 (1.55-2.97) | 1.93 (0.47-7.85) |
|  | 65 | 1.51 (1.21-1.89) | 3.73 (2.7-5.13) | 4.18 (1.06-16.44) |
|  | 75 | 1.61 (1.27-2.04) | 6.3 (4.57-8.69) | 5.38 (1.36-21.29) |
|  | 85 | 1.67 (1.29-2.16) | 11.47 (8.29-15.85) | 12.82 (3.23-50.93) |
| Comorbidities | Cardiovascular disease | 0.929 (0.846, 1.02) | 1.08 (0.993, 1.18) | 0.931 (0.691, 1.25) |
|  | Pulmonary disease | 0.812 (0.732, 0.901) | 1.1 (1.02, 1.2) | 2.21 (1.69, 2.89) |
|  | Diabetes | 1.11 (0.978, 1.27) | 1.3 (1.19, 1.42) | 1.11 (0.814, 1.51) |
|  | Kidney disease | 1.01 (0.876, 1.17) | 1.24 (1.13, 1.35) | 1.07 (0.795, 1.44) |
|  | Liver disease | 0.884 (0.75, 1.04) | 1.29 (1.15, 1.44) | 1.26 (0.866, 1.84) |
|  | HIV/AIDS | 1.67 (0.681, 4.08) | 1.59 (0.579, 4.36) | 6.79 (0.825, 55.8) |
|  | Cancer | 0.898 (0.773, 1.04) | 0.926 (0.84, 1.02) | 0.912 (0.667, 1.25) |
|  | Obesity | 1.05 (0.949, 1.15) | 1.11 (1.02, 1.2) | 0.853 (0.639, 1.14) |
| Race | White | 1 (Reference) | 1 (Reference) | 1 (Reference) |
|  | Asian | 0.87 (0.739, 1.02) | 1.1 (0.906, 1.33) | 0.335 (0.0822, 1.37) |
|  | Black/African American | 0.911 (0.761, 1.09) | 1.08 (0.878, 1.34) | 0.354 (0.0863, 1.45) |
|  | Native American | 1.18 (0.737, 1.89) | 1.02 (0.569, 1.84) | 1.12 (0.15, 8.3) |
|  | Hawaiian / Pacific | 2.3 (1.15, 4.58) | 1.92 (0.776, 4.74) | 3.45 (0.378, 31.4) |
|  | Other | 0.899 (0.724, 1.12) | 1.08 (0.834, 1.41) | 1.38e-07 (0, Inf) |
|  | Unknown | 1.02 (0.708, 1.46) | 0.589 (0.307, 1.13) | 1.08e-07 (0, Inf) |
| Ethnicity | Hispanic or Latino | 1 (Reference) | 1 (Reference) | 1 (Reference) |
|  | Not Hispanic or Latino | 0.926 (0.791, 1.08) | 0.974 (0.797, 1.19) | 1.03 (0.411, 2.6) |
|  | Unknown | 0.946 (0.728, 1.23) | 0.639 (0.456, 0.897) | 1.09 (0.338, 3.5) |
| Sex | Female | 1 (Reference) | 1 (Reference) | 1 (Reference) |
|  | Male | 1.15 (1.09, 1.22) | 1.2 (1.13, 1.27) | 1.53 (1.26, 1.87) |

**Table S3. Demographic and clinical characteristics of cases and controls for the secondary analysis of BNT162b2 starting after the first vaccine dose.** The underlying population corresponds to the set of individuals who received their first BNT162b2 dose on or after December 15, 2020, had not received any prior doses of COVID-19 vaccines, and had no record of a positive PCR test prior to the first dose of BNT162b2. The eligible population corresponds to the subset of the underlying population who underwent at least one symptomatic test four or more days after their first dose. Cases correspond to the first positive symptomatic test for a given individual in the eligible population; by definition, the number of individuals contributing cases is the same as the number of cases. Controls correspond to negative symptomatic tests after which occur before the given individual has experienced any positive SARS-CoV-2 PCR tests; an individual can contribute multiple controls during the study period, and so the number of individuals in the control population is less than the total number of tests (controls) contributed. Because an individual can contribute negative tests (controls) prior to contributing a positive test, the number of individuals in the eligible population is smaller than the sum of the number of individuals in the case and control populations. Sub-sampling in the control population refers to the process in which negative tests from a given individual were (i) excluded if they occurred after a positive test or within the 15 days prior to a positive test (possible false negative), (ii) randomly sampled if they occurred within 15 days of each other (possibly during the same symptomatic illness), and (iii) randomly sampled if the individual contributed more than three negative tests during the study period. A stratum (defined by the regression equation as a unique combination of county and calendar week of testing) is considered analyzable if it includes at least one case and at least one control, because strata including only cases or only controls do not contribute to the estimation of the regression coefficients. For cases and controls, all summarized characteristics correspond to only individuals who contributed at least one symptomatic test to an analyzable stratum.

| **Characteristic** | **Underlying Population**    1+ dose of BNT162b2 on or after Dec 15, 2020 | **Eligible Population**  1+ dose of BNT162b2 with symptomatic test 4+ days after first dose | **Case Population**  1+ positive symptomatic test 4+ days after first dose | **Control Population**  1+ negative symptomatic test 4+ days after first dose, sub-sampled |
| --- | --- | --- | --- | --- |
| Number of individuals | 254304 | 42529 | 6713 | 37213 |
| Number of symptomatic tests |  | 53069 | 6713 | 46356 |
| Number of symptomatic tests in analyzable strata  *Total*  *Bucketed by time since first vaccine dose*   - Days 4-10 - Days 11-13 - Days 14-20 - Days 21-27 - Days 28-34 - Days 35-41 - Days 42-71 - Days 72-101 - Days 102-131 - Days 132-161 - Days 162-191 - Days 192-221 - Days 222-251 - Days 252-281 - Days 282-311 - Days 312-341 - Days 342-371 - Days 372+ |  |  | 6605  221  89  139  79  50  10  92  148  300  540  750  1153  1304  902  444  275  101  8 | 37791  694  313  781  702  594  580  2427  2335  2954  3962  4959  5973  5667  3500  1426  674  239  11 |
| Number of individuals contributing symptomatic tests in analyzable strata |  |  | 6605 | 31330 |
| Age (years)   - Mean (sd) - 18-24 - 25-34 - 35-44 - 45-54 - 55-64 - 65-74 - 75-84 - 85+ | 50.5 (21.1)  16,747 (6.6%)  29,430 (11.6%)  32,164 (12.6%)  33,576 (13.2%)  45,652 (18.0%)  40,374 (15.9%)  25,575 (10.1%)  7,654 (3.0%) | 52.1 (18.9)  2,948 (6.9%)  6,758 (15.9%)  7,339 (17.3%)  6,175 (14.5%)  7,761 (18.2%)  5,743 (13.5%)  4,106 (9.7%)  1,699 (4.0%) | 50.8 (17.7)  402 (6.1%)  1,033 (15.6%)  1,337 (20.2%)  1,089 (16.5%)  1,256 (19.0%)  778 (11.8%)  525 (7.9%)  185 (2.8%) | 50.9 (18.7)  2,319 (7.4%)  5,461 (17.4%)  5,597 (17.9%)  4,620 (14.7%)  5,607 (17.9%)  3,893 (12.4%)  2,720 (8.7%)  1,113 (3.6%) |
| State of Primary Residence   - Arizona - Florida - Iowa - Minnesota - Wisconsin - Other | 27,691 (10.9%)  32,477 (12.8%)  3,512 (1.4%)  129,741 (51.0%)  48,462 (19.1%)  12,421 (4.9%) | 2,761 (6.5%)  4,465 (10.5%)  278 (0.7%)  22,557 (53.0%)  12,344 (29.0%)  124 (0.3%) | 401 (6.1%)  604 (9.1%)  37 (0.6%)  3,528 (53.4%)  2,035 (30.8%)  0 (0.0%) | 2,008 (6.4%)  3,271 (10.4%)  42 (0.1%)  17,042 (54.4%)  8,967 (28.6%)  0 (0.0%) |
| Sex   - Female - Male - Unknown | 142,471 (56.0%)  111,801 (44.0%)  31 (0.0%) | ​​26,482 (62.3%)  16,046 (37.7%)  1 (0.0%) | 3,947 (59.8%)  2,658 (40.2%)  0 (0.0%) | 19,808 (63.2%)  11,521 (36.8%)  1 (0.0%) |
| Race   - Asian - Black/African American - Native American - Hawaiian/Pacific Islander - White - Other - Unknown | 8,567 (3.4%)  8,370 (3.3%)  850 (0.3%)  287 (0.1%)  222,327 (87.4%)  6,339 (2.5%)  7,564 (3.0%) | 1,316 (3.1%)  1,055 (2.5%)  133 (0.3%)  37 (0.1%)  38,862 (91.4%)  831 (2.0%)  295 (0.7%) | 168 (2.5%)  145 (2.2%)  21 (0.3%)  10 (0.2%)  6,098 (92.3%)  118 (1.8%)  45 (0.7%) | 1,064 (3.4%)  829 (2.6%)  101 (0.3%)  25 (0.1%)  28,443 (90.8%)  644 (2.1%)  224 (0.7%) |
| Ethnicity   - Hispanic or Latino - Not Hispanic or Latino - Unknown | 11,091 (4.4%)  232,261 (91.3%)  10,952 (4.3%) | 1,579 (3.7%)  40,370 (94.9%)  580 (1.4%) | 223 (3.4%)  6,296 (95.3%)  86 (1.3%) | 1,206 (3.8%)  29,688 (94.8%)  436 (1.4%) |
| Comorbidities   - Cancer - Cardiovascular disease - Diabetes - HIV/AIDS - Kidney disease - Liver disease - Obesity - Pulmonary disease | 10,940 (5.1%)  39,612 (18.5%)  11,614 (5.4%)  119 (0.1%)  10,147 (4.7%)  6,761 (3.2%)  21,005 (9.8%)  16,694 (7.8%) | 2,166 (5.3%)  9,317 (23.0%)  2,998 (7.4%)  24 (0.1%)  2,523 (6.2%)  1,546 (3.8%)  5,561 (13.7%)  4,393 (10.8%) | 248 (3.9%)  1,282 (20.2%)  445 (7.0%)  6 (0.1%)  323 (5.1%)  195 (3.1%)  858 (13.5%)  575 (9.1%) | 1,464 (4.9%)  6,547 (22.0%)  2,049 (6.9%)  18 (0.1%)  1,697 (5.7%)  1,097 (3.7%)  3,944 (13.2%)  3,189 (10.7%) |
| First Dose Date   - Earliest - 25th % - Median - 75th % - Latest | 12/1/20  2/1/21  3/13/21  4/21/21  1/31/22 | 12/15/20  1/22/21  3/2/21  4/1/21  12/24/21 | 12/17/20  1/26/21  3/10/21  4/7/21  12/24/21 | 12/15/20  1/21/21  3/3/21  4/1/21  12/23/21 |
| Time between first dose and test   - Minimum - 25th % - Median - 75th % - Maximum |  | 4  95  173  229  378 | 4  161  215  254  378 | 4  113  183  231  376 |

**Table S4. Secondary analysis 2: adjusted odds of symptomatic SARS-CoV-2 infection and non-COVID-19 hospitalization by time since first vaccine dose, split by age group**. Adjusted odds were estimated with linear spline equations derived from conditional logistic regression models fit separately for each outcome. The number of positive events in each age group is provided in the first column. Knots through the 90th percentile of the days since first vaccination for the entire cohort are included.

| **Age Group** | **Time Relative to First Dose** | **Adjusted Odds Ratio (95% CI)** | |
| --- | --- | --- | --- |
|  |  | **Symptomatic Infection** | **Non-COVID-19 Hospitalization** |
| 18-44 years  (2419 infections;  791 hospitalizations) | Day 4 | 1 (Reference) | 1 (Reference) |
|  | Days 10 | 1.44 (0.71-2.92) | 2.13 (0.62-7.36) |
|  | Day 14 | 0.88 (0.48-1.6) | 0.9 (0.33-2.46) |
|  | Day 21  (Expected second dose) | 0.72 (0.4-1.32) | 1.63 (0.62-4.29) |
|  | Day 35  (Expected full vaccination) | 0.18 (0.1-0.34) | 1.63 (0.66-4) |
|  | Day 85 | 0.32 (0.19-0.56) | 1.4 (0.58-3.36) |
|  | Day 135 | 0.47 (0.28-0.78) | 1.08 (0.45-2.61) |
|  | Day 185 | 0.52 (0.31-0.86) | 0.86 (0.35-2.09) |
|  | Day 235 | 0.63 (0.38-1.04) | 0.7 (0.28-1.7) |
|  | Day 285 | 0.63 (0.38-1.05) | 0.45 (0.17-1.15) |
| 45-64 years  (2141 infections;  1306 hospitalizations) | Day 4 | 1 (Reference) | 1 (Reference) |
|  | Days 10 | 1.41 (0.63-3.14) | 0.9 (0.35-2.31) |
|  | Day 14 | 0.92 (0.49-1.73) | 0.84 (0.4-1.78) |
|  | Day 21  (Expected second dose) | 0.46 (0.23-0.95) | 0.75 (0.36-1.54) |
|  | Day 35  (Expected full vaccination) | 0.19 (0.09-0.38) | 0.93 (0.48-1.79) |
|  | Day 85 | 0.24 (0.13-0.44) | 0.61 (0.32-1.17) |
|  | Day 135 | 0.38 (0.22-0.66) | 0.59 (0.3-1.13) |
|  | Day 185 | 0.45 (0.26-0.78) | 0.42 (0.21-0.81) |
|  | Day 235 | 0.49 (0.28-0.83) | 0.21 (0.1-0.41) |
|  | Day 285 | 0.44 (0.25-0.76) | 0.31 (0.15-0.64) |
| 65+ years  (1329 infections,  3472 hospitalizations) | Day 4 | 1 (Reference) | 1 (Reference) |
|  | Days 10 | 0.59 (0.22-1.56) | 0.7 (0.37-1.33) |
|  | Day 14 | 0.93 (0.41-2.11) | 0.79 (0.47-1.33) |
|  | Day 21  (Expected second dose) | 0.39 (0.16-0.94) | 0.78 (0.47-1.28) |
|  | Day 35  (Expected full vaccination) | 0.09 (0.03-0.22) | 0.84 (0.52-1.36) |
|  | Day 85 | 0.16 (0.06-0.41) | 0.7 (0.42-1.15) |
|  | Day 135 | 0.22 (0.09-0.52) | 0.48 (0.29-0.8) |
|  | Day 185 | 0.29 (0.13-0.64) | 0.44 (0.26-0.75) |
|  | Day 235 | 0.33 (0.15-0.73) | 0.31 (0.18-0.54) |
|  | Day 285 | 0.26 (0.11-0.61) | 0.32 (0.18-0.58) |

**Table S5. Demographic and clinical characteristics of cases and controls for the sensitivity analysis of BNT162b2 with stratification on county, time of vaccination, and county-level COVID-19 incidence at the time of testing rather than on county and time of testing.** The underlying population corresponds to the set of individuals who received their first BNT162b2 dose on or after December 15, 2020 and were fully vaccinated per protocol (i.e. with two doses administered 18-28 days apart and with no prior positive SARS-CoV-2 PCR tests before the date of full vaccination). The eligible population corresponds to the subset of the underlying population who underwent at least one symptomatic test after the date of full vaccination. Cases correspond to the first positive symptomatic test for a given individual in the eligible population; by definition, the number of individuals contributing cases is the same as the number of cases. Controls correspond to negative symptomatic tests after full vaccination which occur before the given individual has experienced any positive SARS-CoV-2 PCR tests; an individual can contribute multiple controls during the study period, so the number of individuals in the control population is less than the total number of tests (controls) contributed. Because an individual can contribute negative tests (controls) prior to contributing a positive test, the number of individuals in the eligible population is smaller than the sum of the number of individuals in the case and control populations. Sub-sampling in the control population refers to the process in which negative tests from a given individual were (i) excluded if they occurred after a positive test or within the 15 days before a positive test (possible false negative), (ii) randomly sampled if they occurred within 15 days of each other (possibly during the same symptomatic illness), and (iii) randomly sampled if the individual contributed more than three negative tests during the study period. A stratum (defined by the regression equation as a unique combination of county and calendar week of testing) is considered analyzable if it includes at least one case and at least one control, because strata including only cases or only controls do not contribute to the estimation of the regression coefficients. For cases and controls, all summarized characteristics correspond to only individuals who contributed at least one symptomatic test to an analyzable stratum.

| **Characteristic** | **Underlying Population**    Fully vaccinated per-protocol | **Eligible Population**  Fully vaccinated per-protocol with subsequent symptomatic test | **Case Population**  1+ positive symptomatic test after full vaccination | **Control Population**  1+ negative symptomatic test after full vaccination, sub-sampled |
| --- | --- | --- | --- | --- |
| Number of individuals | 219399 | 38622 | 6081 | 33800 |
| Number of symptomatic tests |  | 47525 | 6081 | 41444 |
| Number of symptomatic tests in analyzable strata  *Total*  *Bucketed by time since full vaccination date*   - Days 0-29 - Days 30-59 - Days 60-89 - Days 90-119 - Days 120-149 - Days 150-179 - Days 180-209 - Days 210-239 - Days 240-269 - Days 270-299 - Days 300-329 - Days 330-359 |  |  | 6024     73  127  271  491  691  1070  1343  1006  491  311  131  19 | 39703     3749  3450  3864  4640  5322  5994  5894  3946  1717  789  305  33 |
| Number of individuals contributing symptomatic tests in analyzable strata |  |  | 6024 | 32299 |
| Age (years)   - Mean (sd) - 18-24 - 25-34 - 35-44 - 45-54 - 55-64 - 65-74 - 75-84 - 85+ | 51.1 (21.0)  13,319 (6.1%)  24,890 (11.3%)  27,570 (12.6%)  29,121 (13.3%)  39,752 (18.1%)  35,737 (16.3%)  23,066 (10.5%)  6,910 (3.1%) | 52.0 (18.8)  2,614 (6.8%)  6,229 (16.1%)  6,725 (17.4%)  5,636 (14.6%)  7,074 (18.3%)  5,137 (13.3%)  3,683 (9.5%)  1,524 (3.9%) | 50.8 (17.7)  354 (5.9%)  958 (15.9%)  1,220 (20.3%)  992 (16.5%)  1,158 (19.2%)  690 (11.5%)  482 (8.0%)  170 (2.8%) | 52.1 (19.0)  2,179 (6.7%)  5,263 (16.3%)  5,553 (17.2%)  4,637 (14.4%)  5,864 (18.2%)  4,324 (13.4%)  3,147 (9.7%)  1,332 (4.1%) |
| State of Primary Residence   - Arizona - Florida - Iowa - Minnesota - Wisconsin - Other | 24,294 (11.1%)  27,658 (12.6%)  2,806 (1.3%)  111,664 (50.9%)  42,828 (19.5%)  10,149 (4.6%) | 2,577 (6.7%)  4,071 (10.5%)  244 (0.6%)  20,287 (52.5%)  11,337 (29.4%)  106 (0.3%) | 375 (6.2%)  544 (9.0%)  39 (0.6%)  3,165 (52.5%)  1,898 (31.5%)  3 (0.0%) | 2,223 (6.9%)  3,480 (10.8%)  94 (0.3%)  17,136 (53.1%)  9,363 (29.0%)  3 (0.0%) |
| Sex   - Female - Male - Unknown | 123,160 (56.1%)  96,215 (43.9%)  23 (0.0%) | 24,180 (62.6%)  14,442 (37.4%)  0 (0.0%) | 3,609 (59.9%)  2,415 (40.1%)  0 (0.0%) | 20,376 (63.1%)  11,923 (36.9%)  0 (0.0%) |
| Race   - Asian - Black/African American - Native American - Hawaiian/Pacific Islander - White / Caucasian - Other - Unknown | 7,557 (3.4%)  6,686 (3.0%)  659 (0.3%)  241 (0.1%)  192,808 (87.9%)  5,221 (2.4%)  6,227 (2.8%) | 1,216 (3.1%)  932 (2.4%)  110 (0.3%)  34 (0.1%)  35,321 (91.5%)  746 (1.9%)  263 (0.7%) | 153 (2.5%)  128 (2.1%)  16 (0.3%)  8 (0.1%)  5,579 (92.6%)  101 (1.7%)  39 (0.6%) | 1,067 (3.3%)  799 (2.5%)  91 (0.3%)  26 (0.1%)  29,455 (91.2%)  639 (2.0%)  222 (0.7%) |
| Ethnicity   - Hispanic or Latino - Not Hispanic or Latino - Unknown | 9,086 (4.1%)  201,144 (91.7%)  9,169 (4.2%) | 1,394 (3.6%)  36,703 (95.0%)  525 (1.4%) | 201 (3.3%)  5,747 (95.4%)  76 (1.3%) | 1,164 (3.6%)  30,689 (95.0%)  446 (1.4%) |
| Comorbidities   - Cancer - Cardiovascular disease - Diabetes - HIV/AIDS - Kidney disease - Liver disease - Obesity - Pulmonary disease | 9,797 (5.2%)  35,088 (18.8%)  10,309 (5.5%)  103 (0.1%)  9,084 (4.9%)  5,905 (3.2%)  18,497 (9.9%)  14,508 (7.8%) | 1,912 (5.2%)  8,338 (22.6%)  2,665 (7.2%)  21 (0.1%)  2,244 (6.1%)  1,357 (3.7%)  4,992 (13.5%)  3,942 (10.7%) | 220 (3.8%)  1,147 (19.9%)  391 (6.8%)  5 (0.1%)  291 (5.0%)  174 (3.0%)  760 (13.2%)  520 (9.0%) | 1,662 (5.4%)  7,104 (23.1%)  2,250 (7.3%)  18 (0.1%)  1,946 (6.3%)  1,165 (3.8%)  4,208 (13.7%)  3,403 (11.1%) |
| Dates of full vaccination   - Earliest - 25th % - Median - 75th % - Latest | 1/4/21 0:00  3/5/21 0:00  4/14/21 0:00  5/17/21 0:00  1/31/22 0:00 | 1/18/21 0:00  2/25/21 0:00  4/5/21 0:00  5/5/21 0:00  12/28/21 0:00 | 1/20/21 0:00  3/1/21 0:00  4/12/21 0:00  5/10/21 0:00  12/28/21 0:00 | 1/18/21 0:00  2/24/21 0:00  4/1/21 0:00  5/4/21 0:00  12/28/21 0:00 |
| Time between full vaccination and test   - Minimum - 25th % - Median - 75th % - Maximum |  | 0  86  149  197  343 | 1  144  186  222  343 | 0  82  143  193  340 |

**Table S6. Sensitivity analysis: conditional logistic regression with stratification on county, time of vaccination, and county-level COVID-19 incidence at the time of testing rather than on county and time of testing.** There were 6024 positive symptomatic tests, 5728 non-COVID-19 hospitalizations, and 454 cases of non-COVID-19 pneumonia that contributed to analyzable strata after the date of full vaccination. Adjusted odds were estimated with linear spline equations (for time since full vaccination and age) or by exponentiating the coefficients derived from conditional logistic regression models fit separately for each outcome. For “Time Relative to Full Vaccination”, knots through the 90th percentile of the days since full vaccination for the entire cohort are included.

|  |  | **Adjusted Odds Ratio (95% CI)** | | |
| --- | --- | --- | --- | --- |
| **Covariate** | **Level/Category** | **Symptomatic Infection**  **[N = 6024 events]** | **Non-COVID-19 Hospitalization**  **[N = 5728 events]** | **Non-COVID-19 Pneumonia**  **[N = 454 events]** |
| Days Since Full vaccination | Day 0 | 1 (Reference) | 1 (Reference) | 1 (Reference) |
|  | Day 50 | 4.13 (2.5-6.82) | 0.97 (0.82-1.14) | 0.94 (0.5-1.77) |
|  | Day 100 | 7.73 (4.99-11.97) | 0.86 (0.73-1.01) | 0.76 (0.41-1.4) |
|  | Day 150 | 11.68 (7.42-18.39) | 0.72 (0.6-0.88) | 0.61 (0.3-1.25) |
|  | Day 200 | 18 (11.41-28.4) | 0.6 (0.48-0.75) | 0.62 (0.27-1.45) |
|  | Day 250 | 22.98 (14.35-36.79) | 0.75 (0.57-0.99) | 0.76 (0.26-2.19) |
| Age | 18 | 1 (Reference) | 1 (Reference) | 1 (Reference) |
|  | 25 | 1.15 (0.88-1.5) | 1.57 (1.04-2.37) | 1.44 (0.17-12.51) |
|  | 35 | 1.42 (1.14-1.77) | 1.11 (0.79-1.57) | 0.61 (0.1-3.64) |
|  | 45 | 1.71 (1.36-2.15) | 1.31 (0.91-1.88) | 1.76 (0.29-10.57) |
|  | 55 | 1.64 (1.31-2.06) | 2.22 (1.58-3.14) | 2.54 (0.46-14.08) |
|  | 65 | 1.53 (1.21-1.93) | 3.8 (2.7-5.34) | 6.49 (1.2-35.09) |
|  | 75 | 1.57 (1.21-2.02) | 6.35 (4.5-8.96) | 8.35 (1.53-45.71) |
|  | 85 | 1.56 (1.19-2.06) | 12.45 (8.81-17.59) | 19.53 (3.57-106.8) |
| Comorbidities | Cardiovascular disease | 0.963 (0.875, 1.06) | 1.1 (1.01, 1.2) | 0.911 (0.671, 1.24) |
|  | Pulmonary disease | 0.818 (0.736, 0.91) | 1.08 (0.996, 1.17) | 2.12 (1.64, 2.75) |
|  | Diabetes | 1.11 (0.977, 1.27) | 1.31 (1.19, 1.43) | 1.12 (0.827, 1.51) |
|  | Kidney disease | 0.989 (0.852, 1.15) | 1.26 (1.15, 1.38) | 1.13 (0.85, 1.51) |
|  | Liver disease | 0.903 (0.764, 1.07) | 1.22 (1.09, 1.36) | 1.2 (0.827, 1.74) |
|  | HIV/AIDS | 2.07 (0.834, 5.13) | 1.47 (0.528, 4.09) | 3.16 (0.373, 26.7) |
|  | Cancer | 0.882 (0.758, 1.03) | 0.913 (0.83, 1) | 0.982 (0.722, 1.34) |
|  | Obesity | 1.05 (0.947, 1.16) | 1.1 (1.01, 1.19) | 0.904 (0.681, 1.2) |
| Race | White | 1 (Reference) | 1 (Reference) | 1 (Reference) |
|  | Asian | 0.831 (0.702, 0.984) | 1.03 (0.849, 1.26) | 0.324 (0.0792, 1.32) |
|  | Black/African American | 0.854 (0.709, 1.03) | 1.12 (0.903, 1.39) | 0.382 (0.0911, 1.6) |
|  | Native American | 1.06 (0.64, 1.77) | 1.3 (0.739, 2.3) | 0.803 (0.0962, 6.7) |
|  | Native Hawaiian / Pacific Islander | 1.99 (0.928, 4.25) | 1.81 (0.714, 4.57) | 1.82 (0.161, 20.6) |
|  | Other | 0.89 (0.712, 1.11) | 0.942 (0.721, 1.23) | 5.11e-08 (0, Inf) |
|  | Unknown | 1.06 (0.732, 1.53) | 0.61 (0.316, 1.17) | 6.8e-08 (0, Inf) |
| Ethnicity | Not Hispanic or Latino | 1 (Reference) | 1 (Reference) | 1 (0.399, 2.51) |
|  | Hispanic or Latino | 0.943 (0.802, 1.11) | 0.948 (0.774, 1.16) | 0.963 (0.299, 3.1) |
|  | Unknown | 0.929 (0.714, 1.21) | 0.637 (0.453, 0.895) |  |
| Sex | Female | 1 (Reference) | 1 (Reference) | 1 (Reference) |
|  | Male | 1.19 (1.12, 1.26) | 1.2 (1.13, 1.27) | 1.49 (1.22, 1.82) |
| Dominant variant | Alpha | 1 (Reference) | 1 (Reference) | 1 (Reference) |
|  | Delta | 1.43 (1.05, 1.96) | 0.996 (0.855, 1.16) | 1.17 (0.672, 2.04) |
|  | Omicron | 1.96 (1.38, 2.79) | 0.879 (0.624, 1.24) | 1.84 (0.563, 6.02) |
|  | Neither | 1.15 (0.652, 2.04) | 0.856 (0.688, 1.07) | 0.729 (0.269, 1.97) |
|  | Unknown | 1.17 (0.835, 1.64) | 1.11 (0.97, 1.27) | 1.19 (0.73, 1.94) |

**Table S7.** **Summary statistics for conditional logistic regression models.** Each model is defined by a unique combination of Stratification Type (date of testing versus date of vaccination), reference timepoint (first dose versus full vaccination), and outcome (symptomatic infection versus non-COVID-19 hospitalization versus non-COVID-19 pneumonia. For each model, the Nagelkerke R-squared value (also known as a pseudo R-squared value) is shown along with the maximum R-squared value. Data shown corresponds to the analyses of individuals of all ages.

| **Stratification Type** | **Time Since**  **First Dose or Full Vax** | **Outcome** | **Pseudo-R^2** | **Max R^2** |
| --- | --- | --- | --- | --- |
| Date of PCR Test | First Dose | Non-COVID-19 hospitalization | 0.106 | 0.666 |
| Date of PCR Test | First Dose | Non-COVID-19 pneumonia | 0.023 | 0.145 |
| Date of PCR Test | First Dose | Symptomatic SARS-CoV-2 infection | 0.014 | 0.707 |
| Date of PCR Test | Full Vax | Non-COVID-19 hospitalization | 0.103 | 0.654 |
| Date of PCR Test | Full Vax | Non-COVID-19 pneumonia | 0.024 | 0.147 |
| Date of PCR Test | Full Vax | Symptomatic SARS-CoV-2 infection | 0.009 | 0.721 |
| Date of Vaccination | Full Vax | Non-COVID-19 hospitalization | 0.067 | 0.647 |
| Date of Vaccination | Full Vax | Non-COVID-19 pneumonia | 0.011 | 0.102 |
| Date of Vaccination | Full Vax | Symptomatic SARS-CoV-2 infection | 0.024 | 0.618 |

**Table S8. Variance inflation factors for conditional logistic regression covariates.** VIFs are used to assess multicollinearity between independent variables in each regression model. A VIF greater than or equal to 5 was considered as evidence that the given covariate showed a concerning degree of multicollinearity with at least one other variable. Data shown corresponds to the analyses of individuals of all ages.

|  | **Stratified on Date of PCR Test** | | **Stratified on Date of Vaccination** |
| --- | --- | --- | --- |
| **Covariate** | **1 dose** | **2 dose** | **2 dose** |
| Age | 1.24 | 1.24 | 1.28 |
| Comorbidity - Cancer | 1.17 | 1.16 | 1.18 |
| Comorbidity - Cardiovascular disease | 2.05 | 2.04 | 2.11 |
| Comorbidity - Diabetes | 1.46 | 1.46 | 1.47 |
| Comorbidity - HIV / AIDS | 1 | 1 | 1 |
| Comorbidity - Kidney disease | 1.38 | 1.38 | 1.41 |
| Comorbidity - Liver disease | 1.13 | 1.13 | 1.13 |
| Comorbidity - Obesity | 1.52 | 1.51 | 1.52 |
| Comorbidity - Pulmonary disease | 1.31 | 1.3 | 1.32 |
| County-level COVID-19 cases per 100K | NA | NA | 1.51 |
| Days since 1st vaccine dose | 1.01 | NA | NA |
| Days since 2nd vaccine dose | NA | 1.01 | 1.81 |
| Ethnicity - Hispanic or Latino | 1.19 | 1.19 | 1.19 |
| Ethnicity - Unknown | 1.27 | 1.28 | 1.26 |
| Race - Asian | 1.01 | 1.01 | 1.02 |
| Race - Black / African American | 1.01 | 1.01 | 1.01 |
| Race - Native American | 1 | 1 | 1 |
| Race - Native Hawaiian / Pacific Islander | 1 | 1 | 1 |
| Race - Other | 1.19 | 1.19 | 1.19 |
| Race - Unknown | 1.27 | 1.28 | 1.26 |
| Sex - Male | 1.02 | 1.02 | 1.02 |
| Variant - Delta | NA | NA | 3.83 |
| Variant - Neither | NA | NA | 1.19 |
| Variant - Omicron | NA | NA | 1.65 |
| Variant - Uncertain | NA | NA | 2.37 |
